# Genomic analysis of almost 8,000 *Salmonella* genomes reveals drivers and landscape of antimicrobial resistance in China

**DOI:** 10.1128/spectrum.02080-23

**Published:** 2023-10-03

**Authors:** Yanan Wang, Xuebin Xu, Baoli Zhu, Na Lyu, Yue Liu, Sufang Ma, Shulei Jia, Bo Wan, Yongkun Du, Gaiping Zhang, George F. Gao

**Affiliations:** 1 International Joint Research Center of National Animal Immunology, College of Veterinary Medicine, Henan Agricultural University, Zhengzhou, Henan, China; 2 CAS Key Laboratory of Pathogen Microbiology and Immunology, Institute of Microbiology, Chinese Academy of Sciences (CAS), Beijing, China; 3 Longhu Laboratory of Advanced Immunology, Zhengzhou, Henan, China; 4 Department of Microbiology, Shanghai Municipal Center for Disease Control and Prevention, Shanghai, China; 5 Savaid Medical School, University of Chinese Academy of Sciences, Beijing, China; 6 Beijing Key Laboratory of Antimicrobial Resistance and Pathogen Genomics, Beijing, China; 7 Department of Pathogenic Biology, School of Basic Medical Sciences, Southwest Medical University, Luzhou, Sichuan, China; Yangzhou University, Yangzhou, Jiangsu, China

**Keywords:** *Salmonella *database, antimicrobial resistance, *Salmonella*, mobilome, virulome, antibiotic resistance genes, public health, food safety

## Abstract

Foodborne *Salmonella* infection remains a major public health concern worldwide. With rising antimicrobial resistance, genomic surveillance is key to tracking outbreaks and monitoring transmission, but there is no comprehensive national surveillance scheme for *Salmonella* involving humans, food, animals, and the environment in China. Moreover, the association between antimicrobial resistance and climate, social, and economic factors has rarely been investigated. Here, we use 1,962 *Salmonella* isolates collected from 22 Chinese provinces and add 6,035 publicly available genomes to build a Chinese local *Salmonella* genome database version 2 (CLSGDB v2) representing 30 Chinese provinces, covering 1905–2022. Using the CLSGDB v2, we mapped the landscape and spatiotemporal dynamics of antimicrobial resistance markers, virulome, and mobilome in *Salmonella*. We identified 317 *mcr* positive and 745 azithromycin resistance genes positive *Salmonella* isolates out of 7,997 isolates. We further uncovered the geographic distribution veil of *mcr-1*, *fosA7*, *fosA3*, *mph(A)*, and *bla*_CTX-M-55_ genes in China, all of them resistant to the critically important antimicrobials including colistin, fosfomycin, azithromycin, and the third-generation cephalosporins. Interestingly, economic, climatic, and social factors can drive the rise of antimicrobial resistance was observed. Finally, we release the CLSGDB v2 as an open-access database and thus can assist surveillance studies tracking 164 *Salmonella enterica* serovars and 295 sequence types across the globe. The CLSGDB v2 is freely available at https://nmdc.cn/clsgdbv2.

**IMPORTANCE:**

We established the largest Salmonella genome database from China and presented the landscape and spatiotemporal dynamics of antimicrobial resistance genes. We also found that economic, climatic, and social factors can drive the rise of antimicrobial resistance. The Chinese local Salmonella genome database version 2 was released as an open-access database (https://nmdc.cn/clsgdbv2) and thus can assist surveillance studies across the globe. This database will help inform interventions for AMR, food safety, and public health.

## INTRODUCTION

The expanding global demand for animal protein is one of the most noteworthy dietary and consumer trends of our time, which will undoubtedly increase the probability of colonization and spread of foodborne pathogens (e.g., *Salmonella*) as well as antimicrobial resistance (AMR). Recent studies reported that a total of 535,000 cases of non-typhoidal *Salmonella* (NTS) invasive disease occurred, and 14.3 million cases of typhoid and paratyphoid fevers occurred in 2017 around the world (1, 2). In April 2022, World Health Organization reported a multi-country outbreak (from >11 countries) caused by multidrug-resistant (MDR) monophasic *S*. Typhimurium (*S*. I 1,4,[5],12:i:-) ST34 infection linked to food (https://www.who.int/emergencies/disease-outbreak-news/item/2022-DON369), which has brought more attention to food safety and AMR. AMR is one of the greatest global public health threats (3), which has been largely driven by the excessive use of antimicrobials (4). Nowadays, AMR is a leading cause of death around the world, there were an estimated 4.95 million deaths associated with bacterial AMR in 2019, with the highest burdens in low-resource settings (5).

Monitoring longitudinal AMR *Salmonella* data is important not only for analyzing dynamic trends but also for the early identification of emerging resistance genes and superbugs. However, most AMR surveillance studies on *Salmonella* from animals, food, humans, or the environment are limited in their spatial or temporal sampling and mostly focus solely on phenotypic, point-prevalence surveys, or genomic data (6 – 11). Notably, we recently revealed the temporal dynamics of dominant *Salmonella* serovars and AMR in China during 2006–2019 using >35,000 strains and 1,962 whole-genome sequencing (WGS) sequences (named the Chinese local *Salmonella* genome database, CLSGDB), respectively (12). WGS provides a powerful tool for understanding and controlling AMR, genomic epidemiology, and the investigation of outbreaks (7, 8, 13 – 15).

In addition to the significant association between animal antimicrobial consumption and AMR in food-producing animals and between human antimicrobial consumption and AMR, specifically in WHO priority pathogens were observed, positive associations between AMR and several socioeconomic factors, such as population density, average temperature, obesity prevalence, and cattle density, were also reported in the previous study (16). Moreover, increasing local temperature, as well as population density, is associated with increasing antibiotic resistance (percent resistant) in common pathogens, *Escherichia coli*, *Klebsiella pneumoniae*, and *Staphylococcus aureus (* 17, 18). Although *Salmonella* is an important indicator of zoonotic intestinal pathogens, the correlation between antimicrobial resistance of *Salmonella* and socioeconomic factors (including climate, social, and economic factors) has rarely been investigated.

More importantly, the high-quality *Salmonella* genome database involving humans, animals (especially livestock, food animals, and aquatic products), and environmental sources from China based on the “One Health” strategy is lacking. Here, we build the high-quality Chinese local *Salmonella* genome database version 2 (CLSGDB v2) using over 8,000 genomes collected from 30 Chinese provinces between 1905 and 2022 and explore the temporal changes of AMR, virulence genes (VGs), and ARGs, as well as the correlation of climate, social, and economic factors with ARGs, VGs, and mobile genetic elements (MGEs).

## RESULTS

### Construction of the Chinese local *Salmonella* genome database version 2

**Fig 1:**
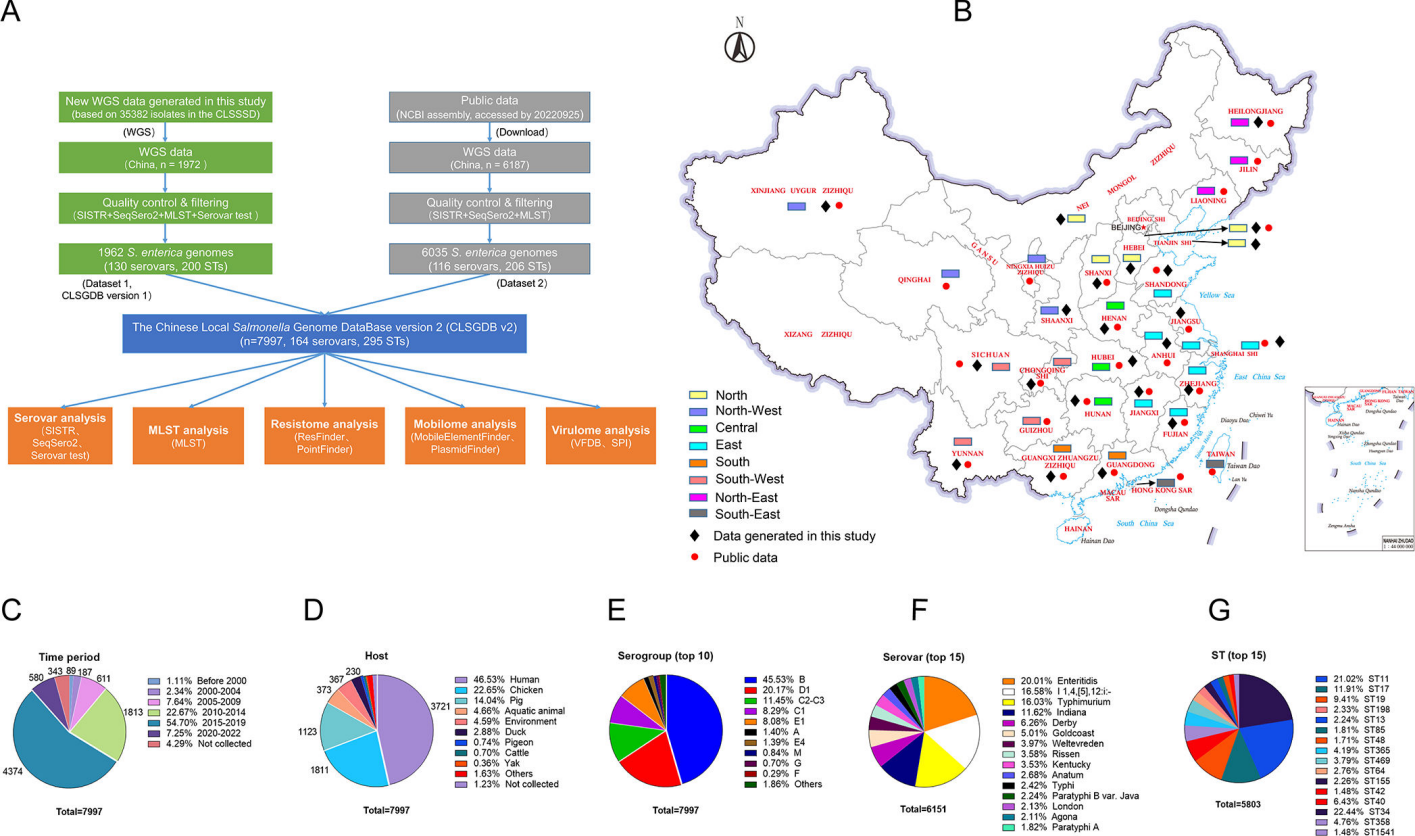
Study design and summary of the Chinese local *Salmonella* genome database version 2 (CLSGDB v2). (A) Flowchart of study design. (B) Origins of the 7,997 *S*. *enterica* isolates from 30 Chinese provinces. Data generated in this study: 22 provinces, Public data: 27 provinces. Base map adapted from Tianditu (https://www.tianditu.gov.cn/), National Platform Common Geospatial Information Services in China, hosted by the Ministry of Natural Resources of the People’s Republic of China. (C–E) All isolates were grouped by sampling (C) time, (D) host, and (E) serogroup. (F) Top 15 serovars among the CLSGDB v2. A total of 164 serovars were detected. (G) Top 15 STs among the CLSGDB v2. A total of 295 STs were detected.

In total, 8,159 *Salmonella* assembly genomes [1,972 from our laboratory and 6,187 from the National Center for Biotechnology Information (NCBI) database as of September 2022] were obtained from human, animal, and environments in 30 Chinese provinces between 1905 and 2022 (Fig. 1A and B; Tables S1 and S2). After quality control and filtering by the combination of serovar prediction (SeqSero2 and SISTR) and multi-locus sequence typing assignment, a total of 7,997 high-quality genomes were obtained (6,035 from NCBI and 1,962 from our laboratory), including 164 serovars and 295 sequence types (STs) (Fig. 1A; Tables S3 to 5). Then, we build the CLSGDB v2 using these 7,997 high-quality genomes. The following analyses are based on the CLSGDB v2.

Of the 6,035 publicly available *Salmonella* genomes, serovar information of 3,425 genomes was recorded. We confirmed that 82.28% (2,818/3,425) of *Salmonella* serovars were correct. We also corrected the serovars of 607 genomes and provided the serovars of 2,610 genomes, accounting for 53.31% of the 6,035 genomes (Table S3). All isolates were chronologically divided into six periods: before 2000 (*n* = 89), 2000–2004 (*n* = 187), 2005–2009 (*n* = 611), 2010–2014 (*n* = 1,813), 2015–2019 (*n* = 4,374), and 2020–2022 (*n* = 580) (Fig. 1C). Isolates were mainly collected from human (*n* = 3,721), chicken (*n* = 1,811), pig (*n* = 1,123), aquatic animal (*n* = 373), environment (*n* = 367), and duck (*n* = 230), accounting for 95.35% of the 7,997 genomes (Fig. 1D; Fig. S1 through S6). A total of 34 serogroups were identified, serogroup B was the most dominant, accounting for 45.5% (Fig. 1E). Of the 164 serovars, the top 15 serovars accounting for 76.92% (Fig. 1F), and 11 serovars were shared among human, chicken, pig, aquatic animal, environment, and duck (Fig. S7). Based on the CLSGDB v2, we confirmed *Salmonella enterica* serovar I 1,4,[5],12:i:- (*S*. I 1,4,[5],12:i:-, a monophasic variant of *S.* Typhimurium) that has emerged as the major cause of human salmonellosis in China.

### Multi-locus sequence type diversity and patterns

**Fig 2:**
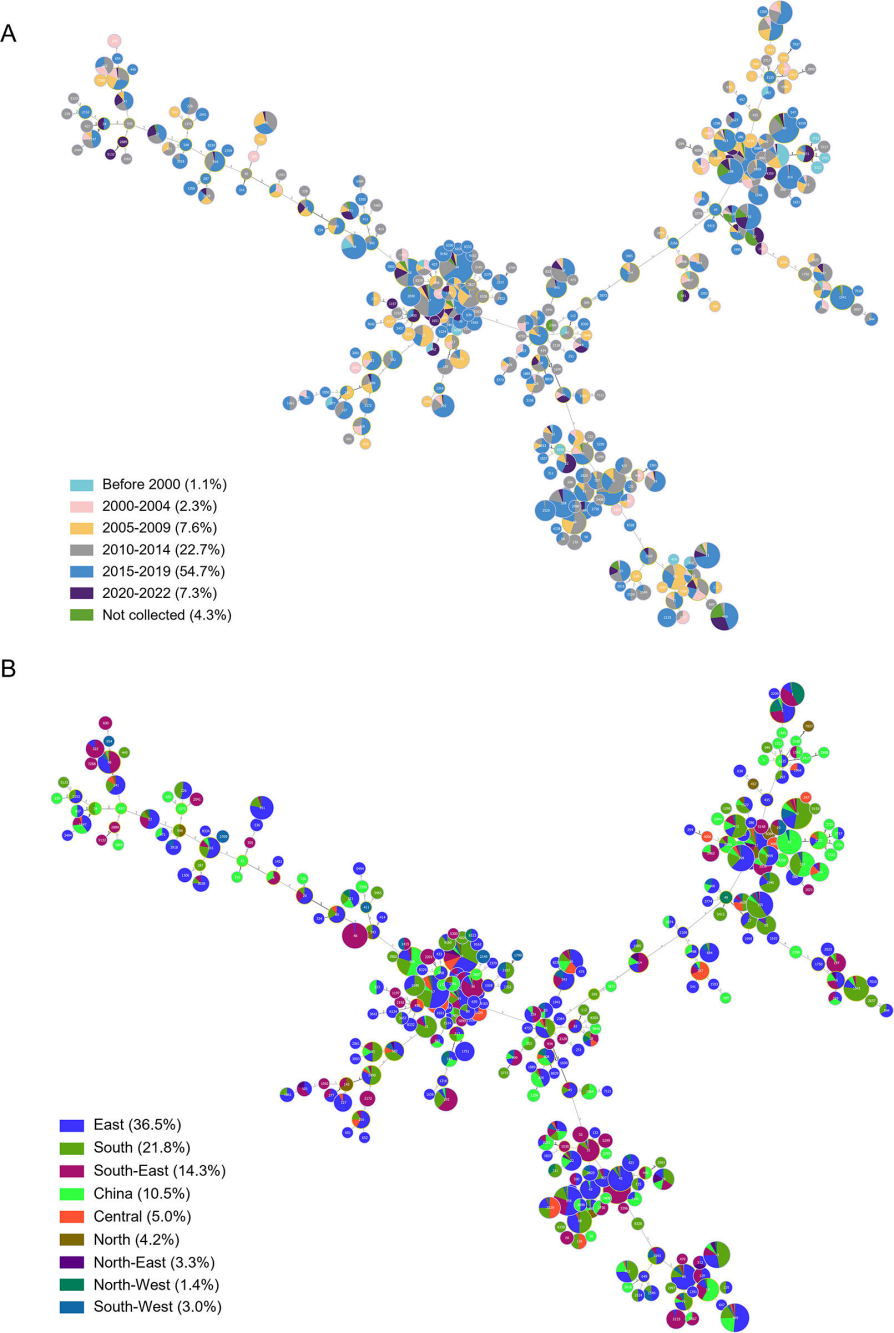
Analysis of the minimum spanning tree of the 7,997 *Salmonella* isolates based on the multi-locus sequence typing. (A and B) The sampling time group (A) and sampling area (B) of all isolates is indicated by different colors. A total of 295 STs were identified. *n* = 7,997.

Multi-locus sequence typing assigned the 7,997 isolates to 295 distinct STs, the top 15 STs accounting for 72.56% (Fig. 1G). 33.90% of the STs (100/295) were represented by a single isolate. ST34 was the most frequently identified and encompassed 1,302 isolates (16.28%) across six chronological data sets (Fig. 2A). Most isolates originated from East (36.5%) and South China (21.8%) (Fig. 2B), where the meat and aquatic production industry and consumption are mainly concentrated. Conversely, ST48 and ST19 were more prevalent before 2000, while ST34, ST11, ST17, and ST40 were mainly observed after 2005–2009 (Fig. S8A). Significant differences were observed in the dominant ST in different animal hosts (Fig. S8B). ST34, ST11, ST17, ST365, and ST34 were frequently identified in pig, chicken, duck, aquatic animal, and human, respectively. However, ST34 was the most frequently identified ST in Central, South, and South-West China, while ST was the most frequently identified ST in North, North-East, East, and North-West China (Fig. S8C). Among the 295 STs, only 10 distinct STs were shared among human, chicken, pig, aquatic animal, environment, and duck (Fig. S9). Briefly, although 295 STs are circulating in different regions of China, we confirmed that the high-risk and highly resistant *S*. I 1,4,[5],12:i:- ST34 is the pandemic ST clone in China.

### Temporal changes of AMR and ARGs

**Fig 3:**
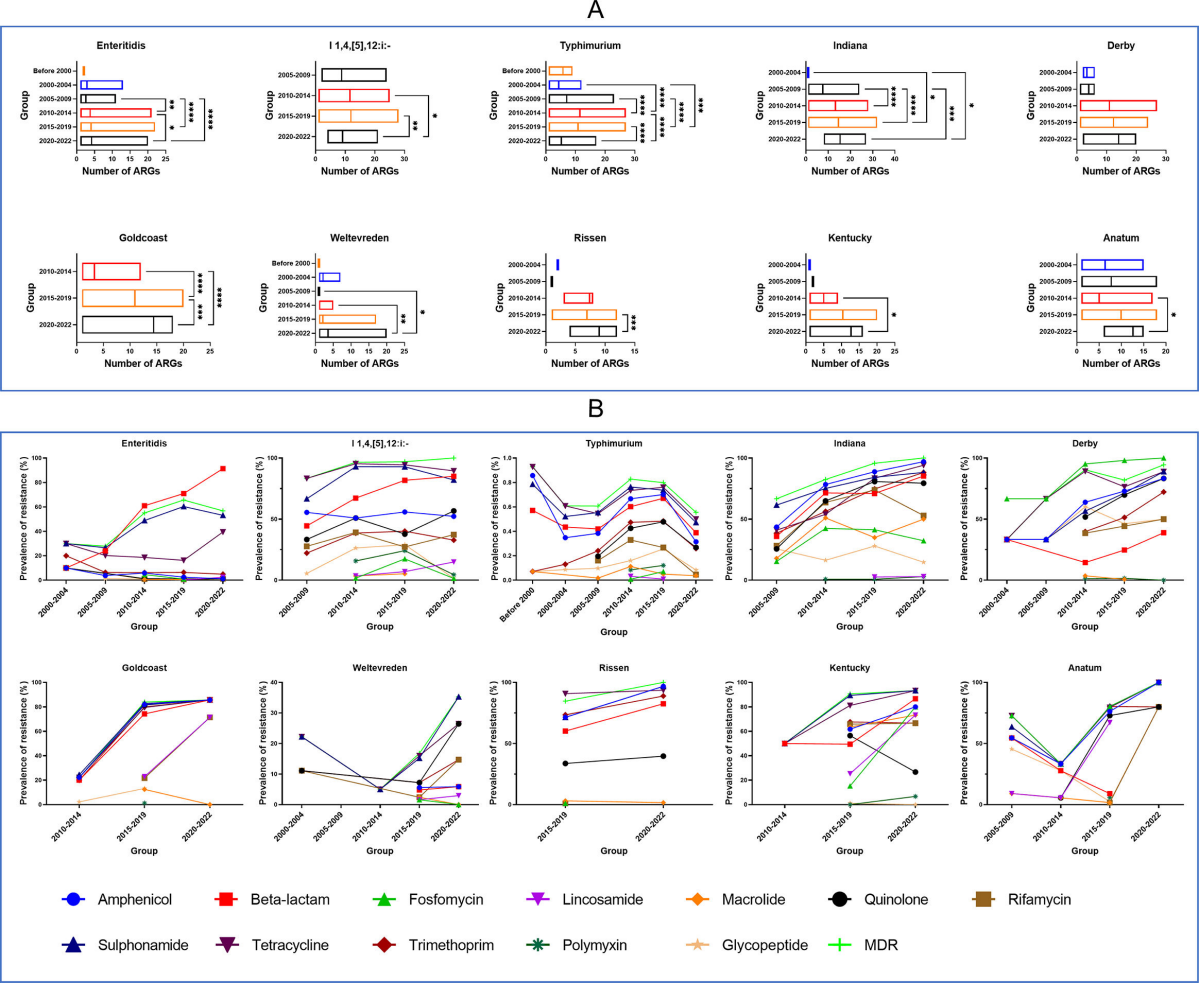
Temporal changes in AMR among different serovars in the CLSGDB v2. (A) Temporal changes in AMR phenotypes among different serovars in the CLSGDB v2 (*n* = 7,997). (B) The distribution of the number of ARGs per isolate among the top 10 serovars in different time groups. The mean ARG count for each group is noted above. The “*” on the right represents *P* values. *: *P* < 0.05; **: *P* < 0.01; ***: *P* < 0.001. ns, no significant difference.

From the genomic analysis, a total of 184 acquired ARGs were identified from the 7,997 *Salmonella* genomes (Table S6). We mainly focused on the AMR trends in the common food animal species, aquatic animal, human, and environmental isolates as well as the top 10 serovars (Fig. S10 and S11; Fig. 3). We observed an increasing trend in AMR during the recent 22-year time span and positive correlations with resistance to critically important antimicrobials with some serovars (Fig. 3). Notably, the trend of AMR *Salmonella* from the environment has shown an alarming rise since 2010. We examined resistances to β-lactams and amphenicols, which are key antimicrobial agents in human and veterinary medicine. The rates of resistance to β-lactam and amphenicol were low in 2000–2004 (20.86% and 19.25%, respectively) but rose rapidly since 2010–2014 (44.24% and 36.18%, respectively) and remained at a high level (62.24% and 49.48%, respectively) in 2020–2022 (Fig. S11). The rates of resistance to β-lactam rose rapidly since 2005–2009 in *S.* Enteritidis, *S*. I 1,4,[5],12:i:-, *S.* Indiana, but decreased in *S.* Anatum (Fig. 3A).

Accordingly, similar trends were observed in the average counts of ARGs per genome. The average counts of ARGs per genome for each sampling group in the CLSGDB v2 were 4.13, 3.44, 3.85, 7.06, 8.34, and 7.81 (Fig. S11). Notably, the number of ARGs per genome in 2020–2022 showed a slight downward trend than 2015–2019, except for chicken and environmental *Salmonella* isolates (Fig. S11). Isolates from pig had a higher prevalence of ARGs than other five groups (Fig. S12), and a lower prevalence of ARGs per genome in 2020–2022 than that in 2015–2019. Among the top 10 serovars, the number of ARGs per genome in 2020–2022 showed a significant downward trend than 2015–2019 in *S.* Typhimurium and *S*. I 1,4,[5],12:i:- was observed (Fig. 3B).

We then tracked the AMR trends in MDR and found the prevalence of MDR rates across geographic regions was distinct. In general, the prevalence of MDR *Salmonella* showed a higher rate in South China than in other regions since 2010–2014 (Fig. S13A). *S*. I 1,4,[5],12:i:- remained at a high MDR level (from 83.33% in 2005–2009 to 100% in 2020–2022). Among these dominant STs, several STs exhibited very high MDR rates (Fig. S13B), for example, ST34 (97.31%), ST17 (89.44%), ST40 (85.52%), ST469 (91.36%), ST198 (96.30%), and ST155 (91.60%).

### Dynamics of ARGs across sampling periods, hosts, serovars, and geographic regions

**Fig 4:**
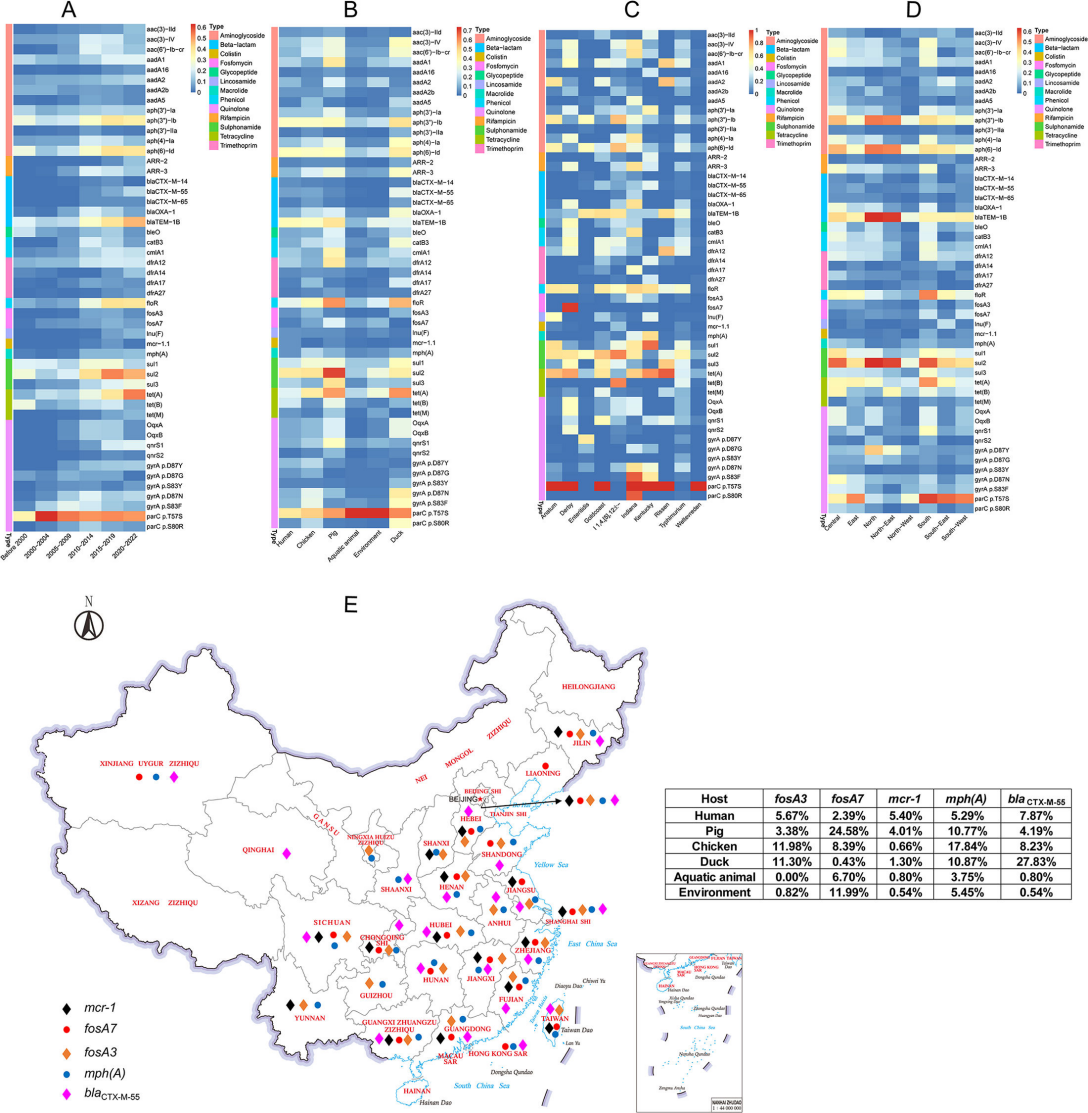
ARG prevalence in the CLSGDB v2. (A–D) ARG prevalence grouped by (A) sampling period, (B) host, (C) serovar, and (D) region in the CLSGDB v2. A total of 184 ARGs were identified in the CLSGDB v2. ARGs that were detected at >3% prevalence (44 ARGs) in *Salmonella* genomes in the CLSGDB v2 are shown. They are clustered by their resistance to different groups of antimicrobial agents and colored by antimicrobial classes on the left. (E) Geographic distribution of colistin ARG *mcr-1*, fosfomycin ARG *fosA3* and *fosA7,* azithromycin ARG *mph(A*) and third-generation cephalosporins ARG *bla*_CTX-M-55_. Base map adapted from Tianditu (https://www.tianditu.gov.cn/), National Platform Common Geospatial Information Services in China, hosted by the Ministry of Natural Resources of the People’s Republic of China.

Then, we analyzed the dynamic trends of individual ARGs detected at >3% prevalence in *Salmonella* genomes in the CLSGDB v2 (Table S6). The prevalence of ARGs conferring resistance to critically important antimicrobials increased between 2000–2004 and 2020–2022, including *bla*_CTX-M-55_ and *bla*_TEM-1B_ (third-generation cephalosporin resistance), *mph(A)* (azithromycin resistance), *qnrS1* (quinolone resistance), *ARR-2* (rifampicin resistance), *floR* (phenicol resistance), and *lnu(F)* (lincosamide resistance). For point mutations, the only significant increase in prevalence was associated with the gyrA mutation D87Y conferring resistance to quinolones from 0.53% to 13.79% (Fig. 4A ; Table S7). The increasing dynamics of specific ARGs in different hosts were observed, for example, *bla*_CTX-M-55_ in chicken, duck, and human, *bla*_TEM-1B_ in pig, human, and the environment (Fig. S14). The prevalence of ARGs varies among different hosts (Fig. 4B). The increasing trend of *bla*_CTX-M-65_ in chicken and human was observed. The *bla*_OXA-1_ increased in the environment while it decreased in the pig. The parC mutation T57S conferring resistance to quinolones showed an increasing trend in chicken and duck while decreasing in humans (Fig. S14). Of the 184 ARGs identified in the CLSGDB v2, 51 ARG types (27.72%) were shared among the common food animal, aquatic animal, human, and the environment (Fig. S15).

We explored the differences and association in the prevalence of individual ARGs across the 10 most common serovars in the CLSGDB v2 (Fig. 4C). Some specific ARGs are detected mainly in one or several serovars with high frequency, for example, fosfomycin resistance gene *fosA7* in *S*. Derby, *mph(A*) in *S*. Kentucky, *S*. Indiana, and *S*. Goldcoast, *mcr-1* in *S.* Typhimurium and *S*. I 1,4,[5],12:i:-. Interestingly, parC mutation T57S conferring resistance to quinolones was detected in 100% of 7 out of 10 dominant serovars, while not found in *S*. Enteritidis, with a very low prevalence of 0.1% and 0.49% in *S.* Typhimurium and *S*. I 1,4,[5],12:i:-, respectively (Fig. 4C). In addition, we also observed the association in the prevalence of individual ARGs and geographic regions (Fig. 4D). For example, *bla*_TEM-1B_ in North and North-East, *floR* and *qnrS1* in South, gyrA mutation D87Y in North and North-East, as well as parC mutation T57S in South, South-East, and South-West. In addition, we mapped the geographical distribution of *mcr-1*, *fosA3*, *fosA7*, *mph(A)*, and *bla*_CTX-M-55_ conferring resistance to the critically important antimicrobials including colistin, fosfomycin, azithromycin, and the third-generation cephalosporins (Fig. 4E). Overall, based on the CLSGDB v2, we mapped the ARG profile of *Salmonella* in China and revealed the dynamic characteristics of ARGs carried by *Salmonella* in different periods, locations, isolation sources, and serovars. To our knowledge, this is the first time to uncover the geographic veil of high-risk mobile resistance genes *mcr-1*, *fosA3*, *fosA7*, *mph(A*), and *bla*_CTX-M-55_ carried by *Salmonella* in China from the genomic view.

### Temporal changes of VGs, plasmids, and MGE profiles

For VG profiles, most isolates carried a large number of VGs (Table S8). No obvious trends or changes in VGs per genome in humans, chickens, pigs, ducks, aquatic animals, and environment were observed (Fig. S16). Significant differences in VGs among the top 10 serovars and geographic regions were observed, respectively (Fig. S17). Among the top 10 serovars, the number of VGs per genome showed a slightly increasing trend (Fig. S18). The typhoid toxin cdtB was identified in 1,752 (21.91% of 7997) genomes distributed in 52 serovars, the top five serovars are Indiana (*n* = 708), Goldcoast (*n* = 307), Typhi (*n* = 148), Paratyphi A (*n* = 111), and Schwarzengrund (*n* = 71).

We also examined the prevalence of SPIs between sampling periods, hosts, geographic regions, and serovars (Fig. S19; Table S9). *Salmonella* genomic island 1 (SGI1) was identified in 263 isolates (20 serovars), accounting for 3.29% of the total. The Yersinia high pathogenicity island was detected in six *S.* Typhimurium and one *S*. Senftenberg genomes in China.

**Fig 5:**
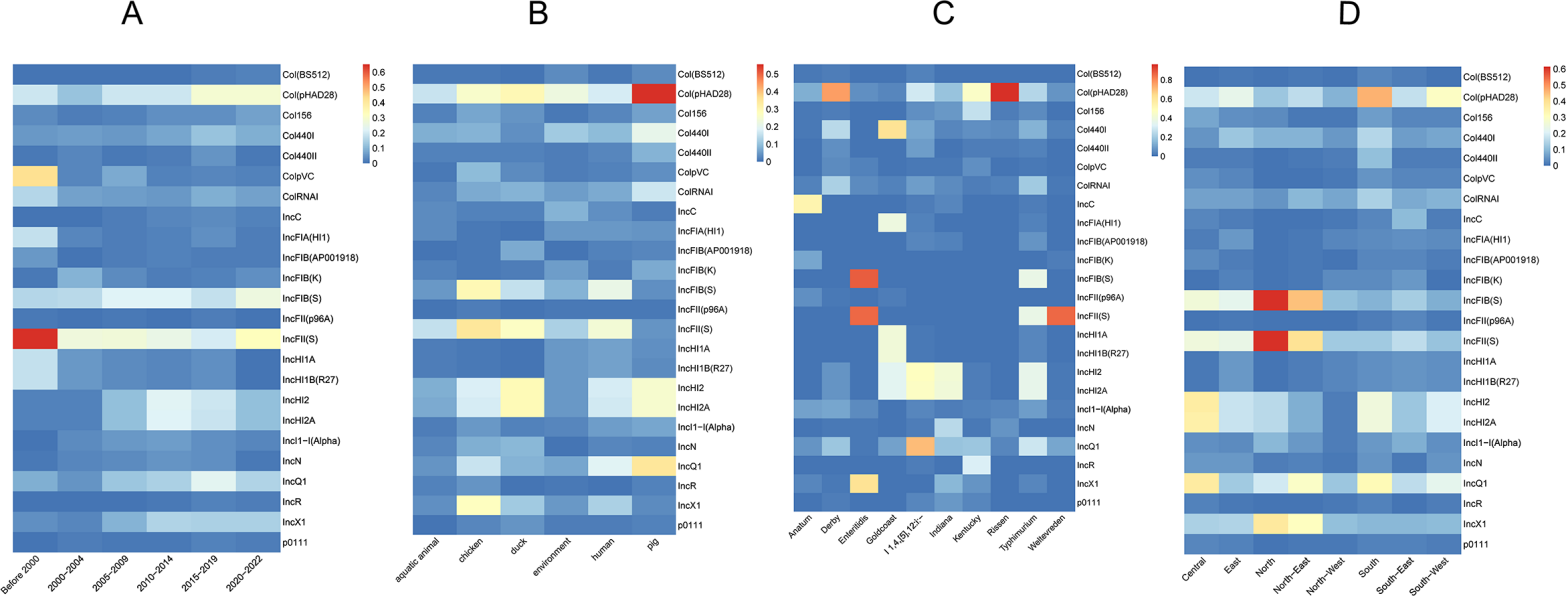
Plasmid replicon prevalence was grouped by sampling periods, hosts, serovars, and regions in the CLSGDB v2. A total of 16923 plasmid replicons classified into 86 types were identified in the CLSGDB v2. Replicons that were detected at >1% prevalence in *Salmonella* genomes in the CLSGDB v2 are shown.

Plasmid replicon analysis also indicated a slight increase in the number of plasmids over time in isolates collected from 2000–2004 to 2015–2019 while a decrease in 2020–2022 (Fig. S20 and S21; Table S10). A total of 16,923 plasmid replicons classified into 86 types were identified in the CLSGDB v2. Isolates from pig had a higher prevalence of plasmid replicons than the other five groups (Fig. S22). The prevalence of several ARG-associated plasmids, such as IncHI2 and IncHI2A, were significantly higher in 2005–2009 than before 2000 (Fig. 5A). In general, the prevalence of dominant plasmid replicon profiles among isolations sources was different (Fig. 5B; Fig. S23). Of the 86 distinct plasmid replicons, a total of 21 types were shared by common food animals, aquatic animals, human, and the environment (Fig. S24). The Col(pHAD28) was dominant in pig, duck, aquatic animal, and environment, while the IncFIB(S) was dominant in human and chicken. Significant differences were observed among isolates from different serovars and geographic regions (Fig. 5C and D). For example, the Col(pHAD28) was dominant in Rissen, Kentucky, and Derby, IncFIB(S) was dominant in *S.* Typhimurium and Enteritidis, while IncQ1 was dominant in *S*. I 1,4,[5],12:i:-.

For MGEs, a total of 50,304 MGEs classified into 1,002 types (Table S11) were identified in the CLSGDB v2. Insertion sequences, MITEEc1, and Tns accounted for 75.50% (*n* = 37,981), 15.47% (*n* = 7,784), and 3.61% (*n* = 1,817). In general, the dominant MGE profiles were similar among isolates from different isolation sources, hosts, and geographic regions (Fig. S25A through S25C). Forty-five MGEs were shared among these common food animal species, aquatic animal, human, and environmental isolates, accounting for 4.49% of the total (Fig. S26). Similar to the plasmid profile in serovars, significant differences in the dominant MGE among different serovars were observed (Fig. S25D).

In addition to plasmids and MGEs, prophages are vital vehicles for horizontal gene transfer in bacteria. Prophages play important roles in the transduction of virulence factors, harboring and transmitting ARGs. Multiple VGs and ARGs were detected on the phage genomes. A total of 6,487 prophages divided into 98 classes were found among the CLSGDB v2, with a maximum of seven intact prophages observed in a single isolate (Table S12). More than half of the isolates (4,196/7,997, 52.47%) harbored at least one intact prophage. *Gifsy_2*, *Salmon_118970_sal3*, and *Gifsy_1* were the three most common prophages (*n* = 1,804, 1,485, and 710, respectively). Compared with the other serovars, *S*. Enteritidis harbored the largest number of intact prophages (Table S13), especially *Gifsy_2* and *Salmon_118970_sal3*. Overall, based on CLSGDBV2, we mined and constructed the mobile genetic element set, the drivers of mobile resistance gene transfer carried by *Salmonella* in China, which expanded our understanding of the overview of ARG helpers prevalent in China, and provided an important resource for further research.

### Correlation analysis of climate, social, and economic factors with ARGs, VGs, and MGEs

**Fig 6:**
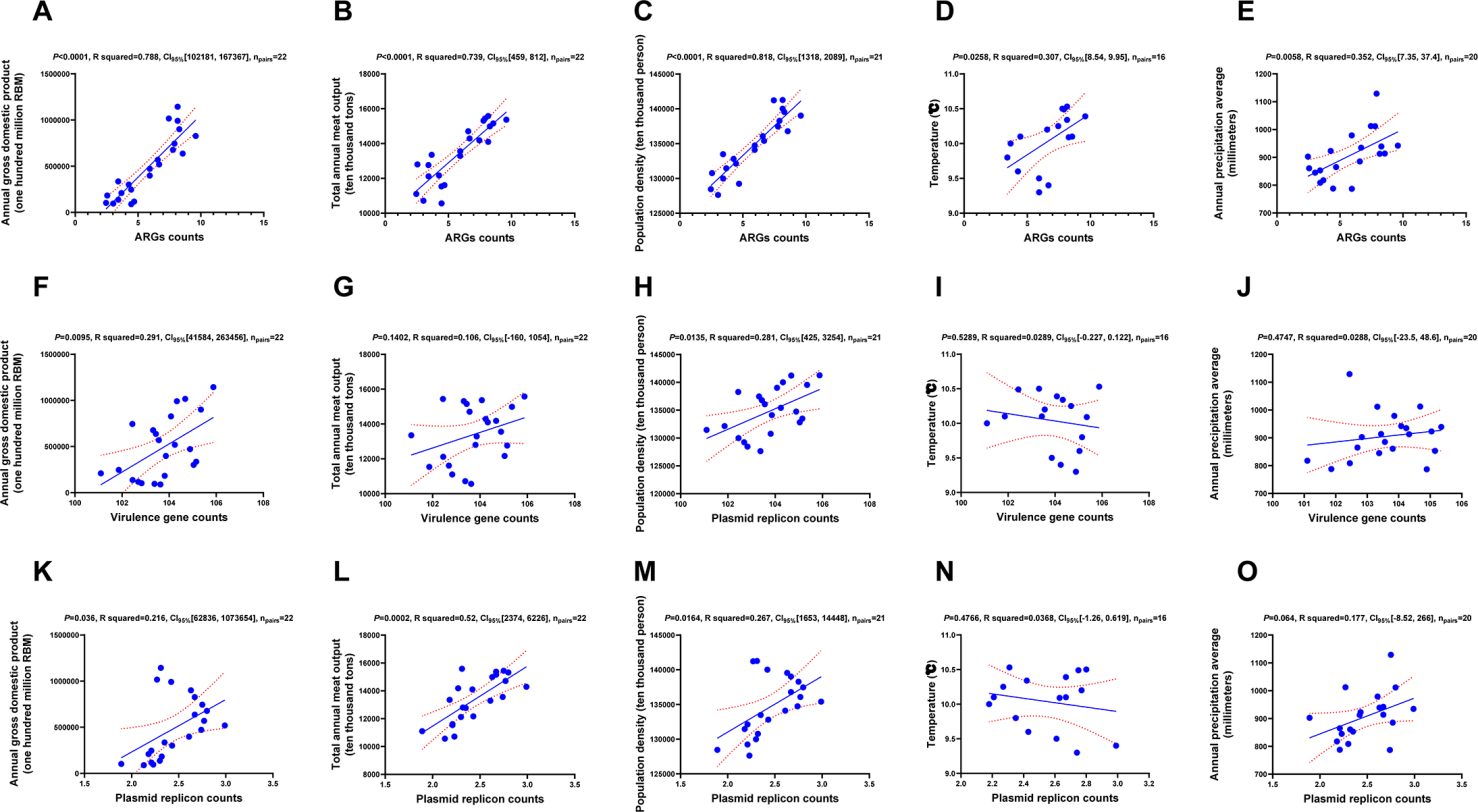
The correlation of climate, social, and economic factors with ARGs, VGs, and MGEs. (A–E) The correlation of the gross domestic product value (A), the gross output of meat (B), population density (C), temperature (D), and precipitation (E) with the average count of ARGs detected from genomes. (F–J) The correlation of the gross domestic product value (F), the gross output of meat (G), population density (H), temperature (I), and precipitation (J) with the average count of plasmid replicons detected from genomes. (K–O) The correlation of the gross domestic product value (K), the gross output of meat (L), population density (M), temperature (N), and precipitation (O) with the average count of VGs detected from genomes. 95% CI, 95% confidence interval.

In general, the gross domestic product value, the gross output of meat, population density, annual mean temperature, and annual mean precipitation were positively correlated with the detected average ARG counts (Fig. 6A through E). The gross domestic product value and population density were positively correlated with the detected average VG counts, while the gross output of meat, annual mean temperature, and annual mean precipitation exhibited no correlation with the detected average VG counts (Fig. 6F through J). The gross domestic product value, the gross output of meat, and population density were positively correlated with the average plasmid-associated replicon counts (Fig. 6K through M), while the annual mean temperature and annual mean precipitation exhibited no correlation with the average plasmid-associated replicon counts (Fig. 6N and O). Moreover, the annual grain output was positively correlated with the detected average ARG counts, average VG counts, and the average plasmid-associated replicon counts in these genomes in the recent 22 years (Fig. S27). We observed a positive association between the number of plasmid replicons, MGEs, and ARGs, and no correlation between ARGs and VGs (Fig. S28).

## DISCUSSION

This study represents the largest *Salmonella* genome database from China and presents the most comprehensive genomic snapshot of *Salmonella* to date, with almost 8,000 high-quality genomes originating from 30 Chinese provinces between 1905 and 2022. Our database also includes 1,962 novel sequenced genomes. Till now, there is no comprehensive national surveillance scheme involving humans, food, animals, and the environment in China for continually monitoring AMR *S. enterica*. We focused on using this data set for documenting the diversity of ARGs, VGs, and MGEs, as well as deciphering evolution patterns and drivers of antimicrobial resistance. In particular, we mapped antimicrobial resistance in chicken, pig, duck, aquatic food animals, humans, and the environment in China during a period of substantial industry growth. To our knowledge, the CLSGDB v2, we established in this study is the largest *Salmonella* genome database in China, and this study is also the first systematic research reporting the genomic characterization of *Salmonella* isolates from human, food, animal, and the environment in China.

The World Health Organization estimated that more than 600 million cases of foodborne diseases (almost 1 in 10 people) and 420,000 deaths could occur every year, particularly children under 5 years of age and people living in low-middle-income countries (https://www.who.int/activities/estimating-the-burden-of-foodborne-diseases). *Salmonella* and *Campylobacter* are some of the most common foodborne pathogens in China, as in other countries. Data from the National Foodborne Disease Outbreak Surveillance System in 2020 showed that *Salmonella* (286 outbreaks and 3,446 illnesses) were the most common bacterial pathogen associated with outbreaks and illnesses, followed by *Vibrio parahaemolyticus* (128 outbreaks and 1,848 illnesses), and *S. aureus* (75 outbreaks and 954 illnesses) (19). To date, over 2,600 serovars have been reported, *Salmonella* Typhimurium and *S*. I 1,4,[5],12:i:- are the predominant serovars in both humans and animals (12, 20). The CLSGDB v2, we built contains 164 serovars and 295 STs, expanding the diversity and size of serovars and STs, which is a continuation of our previous work (12), and is a good genomic data set resource for public health services.

The power of WGS is being increasingly used to address the public health concern of AMR and outbreak investigation (21 – 26). Based on the CLSGDB v2, we have not only mapped the landscape of antimicrobial resistance trends in the top 10 serovars but also analyzed the spatiotemporal dynamics and drivers of AMR. We also observed that the MDR rate was higher in South China than in other regions. The MDR *S*. I 1,4,[5],12:i:- ST34 clone is currently the dominant endemic strain in China and is a major threat to both humans and animals, which is of great concern.

Quinolone-resistant *Salmonella* and extended-spectrum-beta-lactamase-producing *Enterobacteriaceae* have been listed as high-priority pathogens that pose a serious threat to human and animal health and need for the research of new antimicrobials (27). Generally, an obvious upward trend in quinolone and beta resistance was observed in this study (Fig. S10). Among these beta-lactamase-producing isolates, 4 *bla*_NDM_ (*bla*_NDM-1_, *bla*_NDM-5_, *bla*_NDM-9_, and *bla*_NDM-13_) conferring resistance to carbapenems and 20 *bla*_CTX-M_ genes conferring resistance to the third-generation cephalosporins, were identified, respectively. Although the detection of *bla*_NDM_ genes in *Salmonella* in China has been reported (28 – 30), the prevalence of *bla*_NDM_ genes in *Salmonella* is relatively rare. The most prevalent *bla*_CTX-M_ genotype was *bla*_CTX-M-55_ (*n* = 567), which was similar to previous reports (31 – 33).

Colistin is a last-resort antibiotic for severe infections caused by MDR Gram-negative bacteria. Therefore, it is essential to monitor the prevalence of *mcr* genes in *Salmonella*. Mobile colistin resistance genes *mcr*, particularly *mcr-1*, have been found in *Salmonella* around the world (34 – 39). However, a large-scale nationwide study on *mcr* prevalence and transmission in *Salmonella* isolates in China is still lacking. In this study, we identified 317 *mcr*-positive *Salmonella* isolates out of 7,997 isolates from 30 Chinese provinces from 1905 to 2022 using the CLSGDB v2. Since the *mcr-1* gene is the most dominant colistin resistance gene, we mapped the geographic distribution of *mcr-1* carried by *Salmonella* isolates in China (Fig. 4E), which is an important complement and advance to previous studies (12, 34, 40 – 42).

Due to the spread of MDR or extensively drug-resistant (XDR) pathogens and the lack of development of new antimicrobials active against such MDR and XDR pathogens, fosfomycin has recently been reintroduced into clinical application, particularly ESBL-producing and even carbapenem-resistant *Enterobacteriaceae* (43). Fosfomycin-modifying enzymes (Fos) are the primary mechanisms of fosfomycin resistance in *Enterobacteriaceae* isolates. Our recent paper has summarized and compared the prevalence of *fosA7* in both human and non-human origins (12). Here, we mapped the geographical distribution of both *fosA3* and *fosA7* genes in China.

Although azithromycin resistance genes have been reported in China (44 – 46), a systematic study is still lacking. In this study, we identified 745 azithromycin resistance genes (including *erm(B*), *erm(C*), *erm(F*), *erm(T*), *mph(A)*, and *mph(B*)) positive *Salmonella* isolates out of 7,997 isolates from 30 provinces in China. Among these isolates, 728 were *mph(A*) positive isolates. To our knowledge, this is the first geographic distribution map of the azithromycin resistance gene *mph(A*) carried by *Salmonella* in China.

In recent years, China has been one of the largest producers and consumers of antimicrobials. Recent papers reported that the number of ARGs per isolate showed an increased trend (4, 12, 47) and a significant association between antimicrobial consumption and the enrichment of ARGs was observed. The overall prevalence of AMR profiles and corresponding ARGs, however, did not rise in 2020–2022 in the top 10 serovars but exhibited divergent trends. The emergence of novel mobile ARGs posing serious public health concerns, a variety of MGEs carrying mobile ARGs have been detected from clinical isolates. Notably, we mapped the MGE and virulence factor profiles of 7,997 *Salmonella* genomes and revealed for the first time the diversity and distribution of prophages carried by *Salmonella* in China. Moreover, we also reported that economic, climatic, and social factors could drive the rise of AMR, which is similar to the phenomenon observed in other bacteria (17, 18, 47). We hope that the CLSGDB v2 and correlation analysis strengthen the case for data collection and sharing for the public good, especially WGS, which provides data on the early identification of emerging resistance genes and outbreaks.

We acknowledge several limitations in this study. A few genomes (*n* = 89) were collected before 2000, and some of their collection details (exact province location) were not available. Thirty-three from before 2000 were collected from humans in Taiwan Province and may represent a sampling bias. Moreover, no phenotypic data of antimicrobial susceptibility testing were available. In the CLSGDB v2, genomes were mainly collected from chicken, pig, duck, and aquatic food animals, the number of genomes from other animal-derived was too small, and this needs to be further improved. The CLSGDB v2 will be updated at least every 2 years by screening NCBI, ENA, and Enterobase to identify new *Salmonella* genomes. In addition, we will keep generating new WGS data each year to improve the diversity and representation of the database.

In summary, in this study, we establish a large *Salmonella* genome database version 2 (CLSGDB v2) involving humans, food, animals, and the environment in China and present a large-scale comprehensive analysis of *S. enterica* genomes. We present a spatiotemporal picture of antibiotic resistome, virulome, and mobilome diversity in 30 Chinese provinces based on the CLSGDB v2. We integrated and released a high-quality CLSGDB v2 as an open-access database (https://nmdc.cn/clsgdbv2). This database will assist future genomic surveillance studies and will help inform interventions and control for foodborne diseases, AMR, food safety, and public health.

## MATERIALS AND METHODS

### Data set

In this study, *Salmonella* genomes were derived from two data sets. Data set 1 included 1,972 *S*. *enterica* genome sequences, and all details about study design and metadata were described in our previous study (12). In short, data set 1 comprised 1,962 high-quality *S. enterica* genomes, including 130 *Salmonella* serovars from 22 Chinese provinces between 1982 and 2019 (Table S1). These serovars were confirmed by the combination of serological assays and WGS prediction based on genome sequences. Serological tests were carried out according to the Kauffmann-White Scheme by slide agglutination with commercial antiserum for each *Salmonella* strain.

To complete the CLSGDB v2, we screened and summarized the metadata of publicly available *Salmonella* assembly genomes from the National Center of Biotechnology Information BioSample (NCBI BioSample, https://www.ncbi.nlm.nih.gov/biosample/, as of 25 September 2022). A total of 6,187 publicly available *Salmonella* assembly genomes from human, animal, aquatic animal, animal food products environment, and other sources from China were obtained from the NCBI assembly (https://www.ncbi.nlm.nih.gov/assembly/). Metadata and accession numbers are shown in Table S2. These genomes were sourced from 27 Chinese provinces between 1905 and 2022. All 6,187 genomes were selected for bioinformatics analysis using the same software and parameters as Data set 1. We extracted the year of isolating, the province of sampling sites, the host animals, the collection date, and the serovar. We recorded samples for each isolate, including collection date (year and sampling period), isolation source (host and isolation source), geographic location (province), and serovars. It is worth noting that a total of 2,669 (43.14%) of the 6,187 genomes did not record serovar information, and all serovar information of these genomes (*n* = 6,187) needs to be reconfirmed. To better analyze the geographical distribution of *S. enterica* in China, 34 provinces were divided into eight different regions on the basis of geographic proximity. Each sample was taken from one animal or animal product, and the same animal and associated product were reclassified into the same animal. Isolates from feces, blood, swab, food product, and so on, from the same isolation source (animal host, human) were classified into the same host group. For example, isolates from pork, swab, and pig feces are classified into the same isolation source. In the following analysis, we focused on genomes from three common food animal species (including chicken, pig, duck, and aquatic animals), humans, and the environment.

### Whole-genome sequencing of short reads

Isolates were prepared by inoculating a single clone overnight at 37℃ and 180 rpm. Detailed methods were described in our recent paper (12). Genomic DNA was extracted from each isolate using the Wizard Genomic DNA Extraction Kit (Promega, Beijing, China), and Illumina Nextera XT DNA Libraries were prepared and sequenced using the Illumina platform to generate 150 bp paired-end reads.

### Quality control, assembly, and annotation of short reads

Trimmomatic v0.36 (https://github.com/usadellab/Trimmomatic) (48) was used to trim the Illumina sequence reads. Unicycler v0.4.7 (https://github.com/rrwick/Unicycler) (49) was used to assemble genomes. Prokka v1.13.3 (https://github.com/tseemann/prokka) (50) was used to annotate the genome sequences.

### Sequence typing and construction of the CLSGDB v2

*In silico* serotyping was performed using the *Salmonella* Serotyping by whole-genome sequencing (SeqSero2 v1.1.1, https://github.com/denglab/SeqSero2) (51) and the *Salmonella in silico* typing resource (SISTR v1.1.1, https://github.com/phac-nml/sistr_cmd) (52). A combination of SeqSero2 and SISTR outputs (result match) was used to focus our analysis on *S. enterica*. MLST v2.0 (https://github.com/tseemann/mlst) (53) was used to assign a multi-locus sequence type (MLST) to all isolates based on seven housekeeping genes. The criteria for high-quality genome selection are that both two software (SeqSero2 and SISTR) can predict serovar, and MLST can assign an ST to the genome. In addition, the genome size is between 4M and 6M, and the number of contigs is between 50 and 200. Genomes that did not pass the quality control and filtering threshold in either program were removed from the following analysis (*n* = 162). Ten of the 1,972 and 152 of the 6,187 genomes were excluded. The remaining data were considered high-quality genomes for downstream analysis. Our final data set comprised a total of 7,997 high-quality genomes, including 1,962 WGS data generated in this study and 6,035 public data downloaded from the NCBI, forming the Chinese local *Salmonella* genomes database (CLSGDB v2, https://nmdc.cn/clsgdbv2).

### Detection of AMR genes

ResFinder v4.0 (https://bitbucket.org/genomicepidemiology/resfinder/src/master/) (54) was used to determine acquired antibiotic resistance genes (coverage ≥ 90% and identity ≥ 95%) and putative phenotypes of AMR. *Salmonella*-specific point mutations associated with antimicrobial resistance were screened with the PointFinder database (https://bitbucket.org/genomicepidemiology/pointfinder_db/src/master/, coverage = 100% and identity ≥ 80%) (55).

### Identification of virulence genes and *Salmonella* pathogenic islands

ABRicate v0.9.7 (https://github.com/tseemann/abricate) was used to determine virulence genes carried by the genomes with default parameters. SPIFinder v2.0 (https://bitbucket.org/genomicepidemiology/spifinder/src/master/) (56) was used to determine *Salmonella* pathogenic islands (SPIs, identity ≥ 95% and coverage ≥ 60%).

### Identification of mobile genetic elements and plasmids

MobileElementFinder v1.0.3 (https://bitbucket.org/mhkj/mge_finder/src/master/) (57) was used to determine MGEs (identity ≥ 90% and coverage ≥ 90%) in whole genome sequence data. PlasmidFinder v2.0.1 (https://bitbucket.org/genomicepidemiology/plasmidfinder/src/master/, identity ≥ 80% and coverage ≥ 60%) (58) was used to determine putative plasmids carried by the genomes. PHASTER (http://phaster.ca/) was used to predict phage-like regions in the CLSGDB v2.

### Collection of data on climate, social, and economic factors

To examine the association between climate, social, and economic factors and AMR, VGs, and MGEs, we collected the annual mean temperature, precipitation, population density, gross domestic product value, and gross output of meat. These data were collected from the National Bureau of Statistics of China (http://www.stats.gov.cn/sj/). The annual mean precipitation was calculated using the average of the 31 provincial capitals' precipitation in mainland China. The gross output of meat includes pig, cattle, sheep, poultry meat, and aquatic product output. These data are listed in Table S14.

### Statistical analyses

Statistical significance was considered at a *P* value below 0.05. Pie charts and bars were generated using GraphPad Prism v9.0. Regression analysis was used to determine the correlation between climate, social, and economic factors with ARGs, VGs, and MGEs, respectively. Differences between the numbers of ARGs/VGs/plasmid replicons/MGEs/SPIs per isolate in different decade-specific data sets (before 2000, 2000–2004, 2005–2009, 2010–2014, 2015–2019, and 2020–2022) were assessed by the Kruskal-Wallis test using GraphPad Prism v9.0. Interactions between the sampling period, host, serovar, and geographic regions were used to identify specific dynamics. Venn diagrams were drawn using Venn Diagrams (http://bioinformatics.psb.ugent.be/webtools/Venn/). Heatmaps were plotted using the ImageGP platform (http://www.ehbio.com/ImageGP/) (59). The minimum spanning tree of multi-locus STs was generated using PHYLOViZ 2.0 (http://www.phyloviz.net/) (60).

## Data Availability

All genome assemblies used in this study are available from the NCBI under the BioProject number PRJNA766315 and public databases. Sequence reads generated for this study have been submitted to the China National Microbiology Data Center under the BioProject number NMDC10017893. The publicly available genomes were downloaded from the NCBI assembly (https://www.ncbi.nlm.nih.gov/assembly/), and accession numbers are listed in Table S2. The source code used for the Salmonella genome in the CLSGDB v2 analysis is available in the following repository: https://github.com/NANYW123/Salmonella-genome-database-CLSGDB-v2.git. The CLSGDB v2 related files and sequences can be downloaded at https://nmdc.cn/clsgdbv2.

## SUPPLEMENTAL MATERIAL

Supplemental figures spectrum.02080-23-s0001.pdf Fig. S1 to S28.

Supplemental tables part I spectrum.02080-23-s0002.xlsx Tables S1 to S6.

Supplemental tables part II spectrum.02080-23-s0003.xlsx Tables S7 to S14.

ASM does not own the copyrights to Supplemental Material that may be linked to, or accessed through, an article. The authors have granted ASM a non-exclusive, world-wide license to publish the Supplemental Material files. Please contact the corresponding author directly for reuse.
