# Supplementary material for: Genomic analysis of almost 8,000 Salmonella genomes reveals drivers and landscape of antimicrobial resistance in China
Source: Microbiol Spectr. 2023 Oct 3;11(6):e02080-23. doi: 10.1128/spectrum.02080-23 (PMC10714754; doi:10.1128/spectrum.02080-23)
Supplement: Supplemental figures — Fig. S1 to S28. [file spectrum.02080-23-s0001.pdf]

Supplementary figures for:

Genomic analysis of almost 8,000 *Salmonella* genomes reveals drivers and landscape of antimicrobial resistance in China

Yanan Wang[1,2,3,#], Xuebin Xu[4,#], Baoli Zhu[2,5,6,7], Na Lyu[2], Yue Liu[4], Sufang Ma[2], Shulei Jia[2], Bo Wan[1,3], Yongkun Du[1,3], Gaiping Zhang[3,*], & George F. Gao[2,5,*]

[1]International Joint Research Center of National Animal Immunology, College of Veterinary Medicine, Henan Agricultural University, Zhengzhou, Henan 450046, China.

[2]CAS Key Laboratory of Pathogen Microbiology and Immunology, Institute of Microbiology, Chinese Academy of Sciences (CAS), Beijing 100101, China.

[3]Longhu Laboratory of Advanced Immunology, Zhengzhou 450046, China.

[4]Department of Microbiology, Shanghai Municipal Center for Disease Control and Prevention, Shanghai 200336, China.

[5]Savaid Medical School, University of Chinese Academy of Sciences, Beijing 100049, China.

[6]Beijing Key Laboratory of Antimicrobial Resistance and Pathogen Genomics, Beijing 100101, China.

[7]Department of Pathogenic Biology, School of Basic Medical Sciences, Southwest Medical University, Luzhou, Sichuan 646000, China.

[#]These authors contributed equally to this work.

*E-mail: zhanggaip@126.com (Gaiping Zhang) or gaof@im.ac.cn (George F. Gao)

Supplementary figures

Figure S1. Summary of the 3721 *Salmonella enterica* strains of human origin in the CLSGDB v2.
Figure S2. Summary of the 1811 *Salmonella enterica* strains of chicken origin in the CLSGDB v2.
Figure S3. Summary of the 1123 *Salmonella enterica* strains of pig origin in the CLSGDB v2.
Figure S4. Summary of the 373 *Salmonella enterica* strains of aquatic animal origin in the CLSGDB v2.
Figure S5. Summary of the 230 *Salmonella enterica* strains of duck origin in the CLSGDB v2.
Figure S6. Summary of the 367 *Salmonella enterica* strains of environment origin in the CLSGDB v2.
Figure S7. Shared and unique serovars among aquatic animals, chickens, ducks, pigs, humans, and the environment in the CLSGDB v2.
Figure S8. ST prevalence is grouped by sampling periods (A), hosts (B), and regions (C) in the CLSGDB v2.
Figure S9. Shared and unique STs among aquatic animals, chickens, ducks, pigs, humans, and the environment in the CLSGDB v2.
Figure S10. Temporal changes in AMR phenotypes among different isolation sources in the CLSGDB v2 (n = 7997).
Figure S11. Temporal changes in AMR among different isolation sources in the CLSGDB v2.
Figure S12. The distribution of the number of ARGs per isolate among humans, the different animal host groups, and the environment in the CLSGDB v2.
Figure S13. Differences in MDR rates between (A) geographic regions and (B) STs in the CLSGDB.
Figure S14. ARG prevalence grouped sampling period among different isolation sources in the CLSGDB v2.
Figure S15. Shared and unique ARGs among aquatic animals, chickens, ducks, pigs, humans, and the environment in the CLSGDB v2.
Figure S16. Temporal changes in virulence genes among different isolation sources in the CLSGDB v2.
Figure S17. VG prevalence was grouped by sampling periods, hosts, serovars, and regions in the CLSGDB v2.
Figure S18. The distribution of the number of virulence genes per isolate among the top

10 serovars in different time groups.

Figure S19. SPI prevalence was grouped by sampling periods, hosts, regions, and serovars in the CLSGDB v2.

Figure S20. Temporal changes in plasmid replicons among different isolation sources in the CLSGDB v2.

Figure S21. The distribution of the number of plasmid replicon genes per isolate among the top 10 serovars in different time groups.

Figure S22. The distribution of the number of plasmid replicons per isolate among different hosts.

Figure S23. Plasmid prevalence grouped sampling period among different isolation sources in the CLSGDB v2.

Figure S24. Shared and unique plasmid replicons among aquatic animals, chicken, duck, pig, humans, and environment in the CLSGDB v2.

Figure S25. MGE prevalence was grouped by sampling periods (A), hosts (B), serovars (C), and regions (D) in the CLSGDB v2.

Figure S26. Shared and unique MGEs among aquatic animals, chicken, duck, pig, humans, and environment in the CLSGDB v2.

Figure S27. The correlation of annual grain output with ARGs, VGs, and MGEs.

Figure S28. The correlation of mean counts of ARGs, plasmid replicons, MGEs, and VGs detected from genomes in the CLSGDB v2.

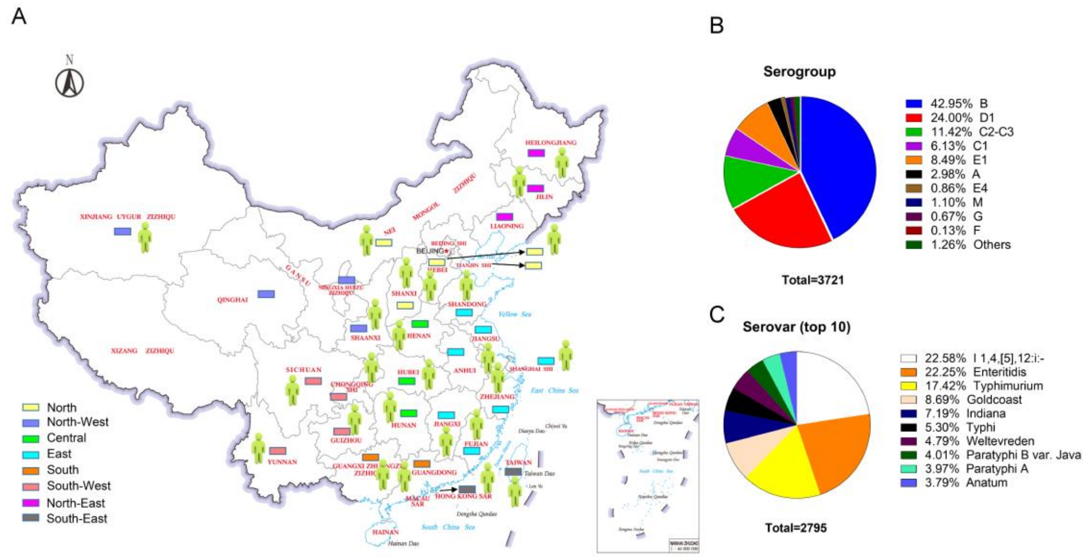

Figure S1. Summary of the 3721 *Salmonella enterica* strains of human origin in the CLSGDB v2.

A, Origins of the 3721 *Salmonella enterica* isolates from 25 Chinese provinces. B, All isolates were grouped by serogroup. C, Top 10 serovars of human origin among the CLSGDB v2. A total of 117 serovars were detected. A total of 188 STs were detected.

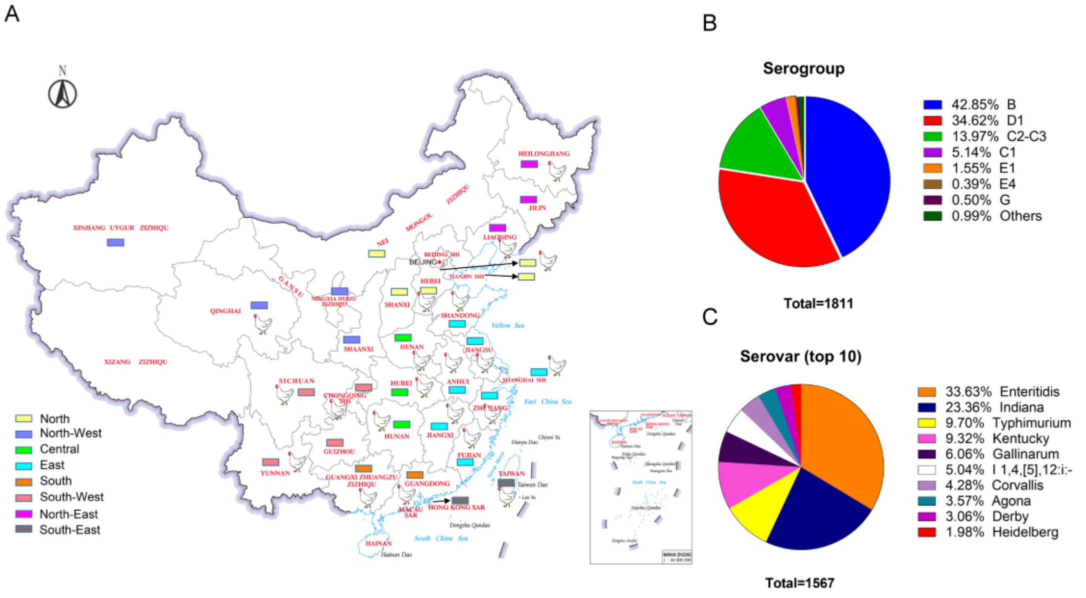

Figure S2. Summary of the 1811 *Salmonella enterica* strains of chicken origin in the CLSGDB v2.

A, Origins of the 1811 *Salmonella enterica* isolates from 20 Chinese provinces. B, All isolates were grouped by serogroup. C, Top 10 serovars of chicken origin among the CLSGDB v2. A total of 58 serovars were detected. A total of 79 STs were detected.

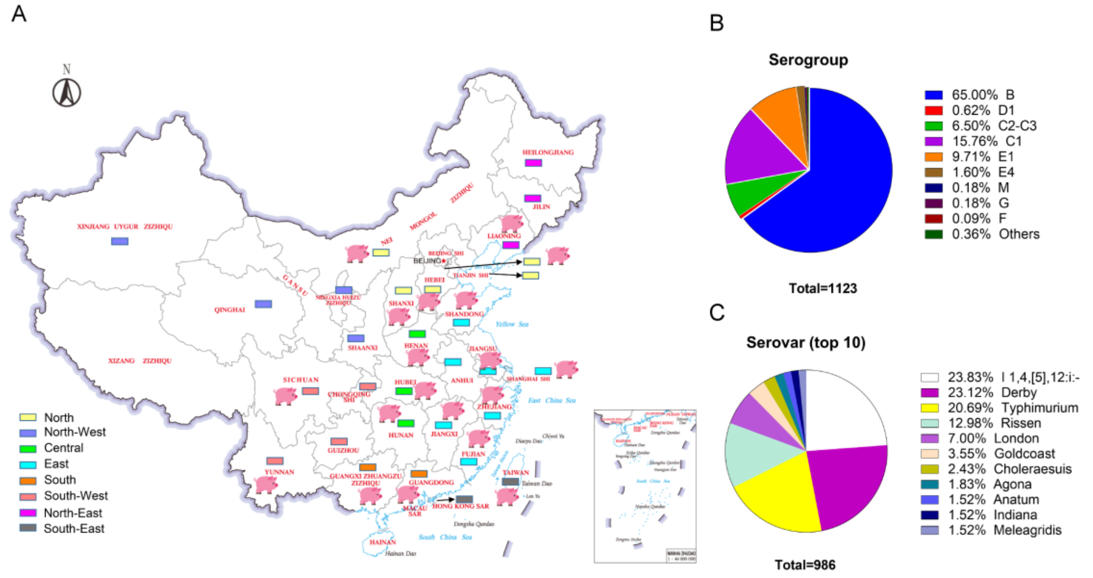

Figure S3. Summary of the 1123 *Salmonella enterica* strains of pig origin in the CLSGDB v2.

A, Origins of the 1123 *Salmonella enterica* isolates from 21 Chinese provinces. B, All isolates were grouped by serogroup. C, Top 10 serovars of pig origin among the CLSGDB v2. A total of 54 serovars were detected. A total of 75 STs were detected.

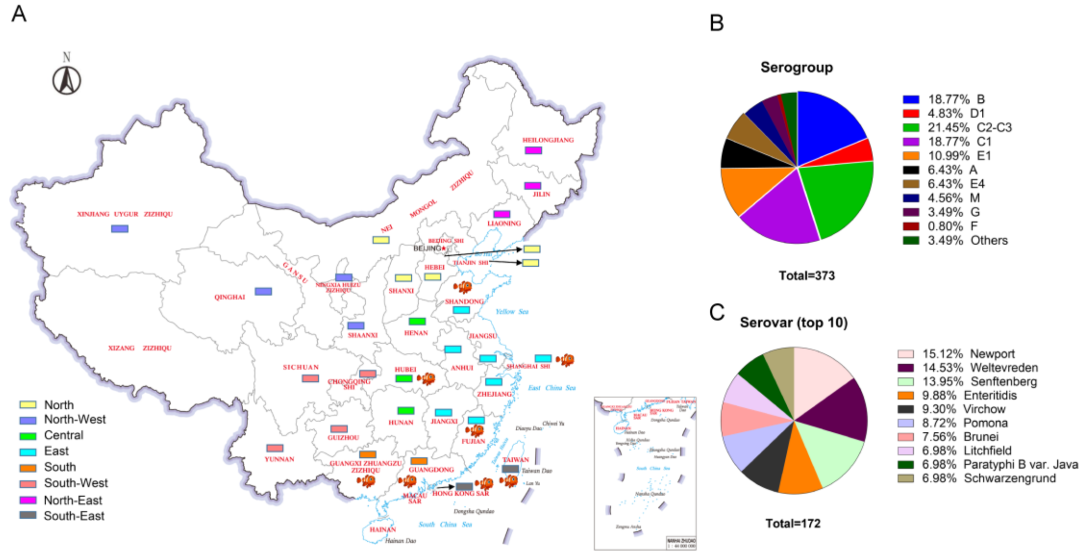

Figure S4. Summary of the 373 *Salmonella enterica* strains of aquatic animal origin in the CLSGDB v2.

A, Origins of the 373 *Salmonella enterica* isolates from 8 Chinese provinces. B, All isolates were grouped by serogroup. C, Top 10 serovars of aquatic animal origin among the CLSGDB v2. A total of 81 serovars were detected. A total of 107 STs were detected.

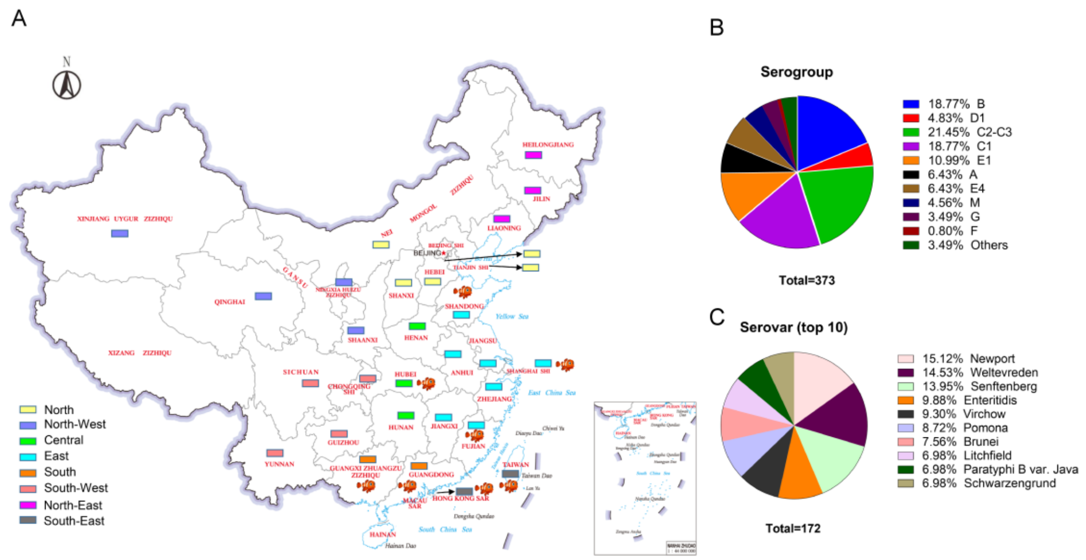

Figure S5. Summary of the 230 *Salmonella enterica* strains of duck origin in the CLSGDB v2.

A, Origins of the 230 *Salmonella enterica* isolates from 13 Chinese provinces. B, All isolates were grouped by serogroup. C, Top 10 serovars of duck origin among the CLSGDB v2. A total of 20 serovars were detected. A total of 25 STs were detected.

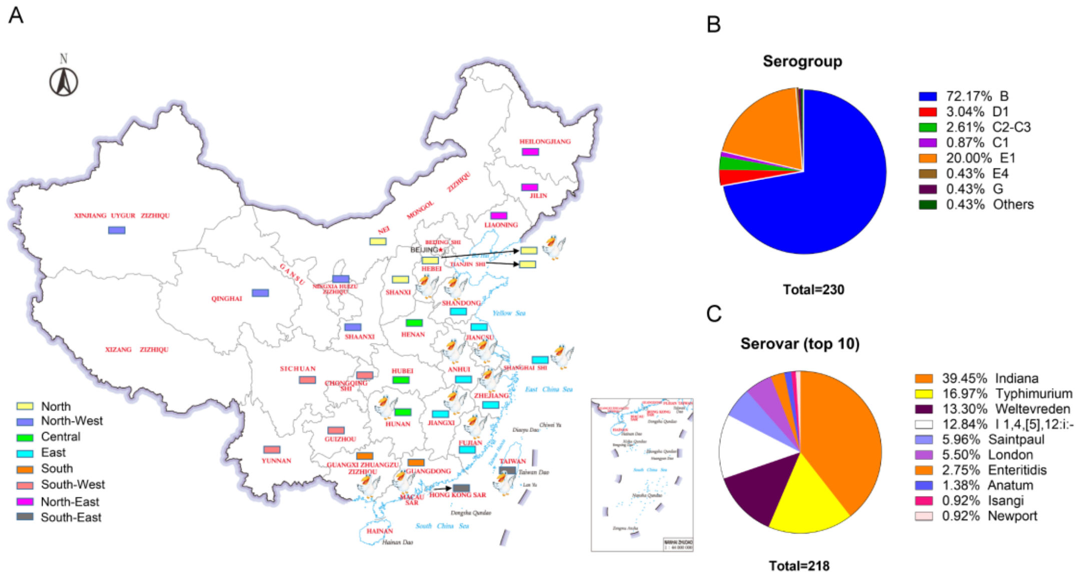

Figure S6. Summary of the 367 *Salmonella enterica* strains of environment origin in the CLSGDB v2.

A, Origins of the 367 *Salmonella enterica* isolates from 13 Chinese provinces. B, All isolates were grouped by serogroup. C, Top 10 serovars of environment origin among the CLSGDB v2. A total of 77 serovars were detected. A total of 98 STs were detected.

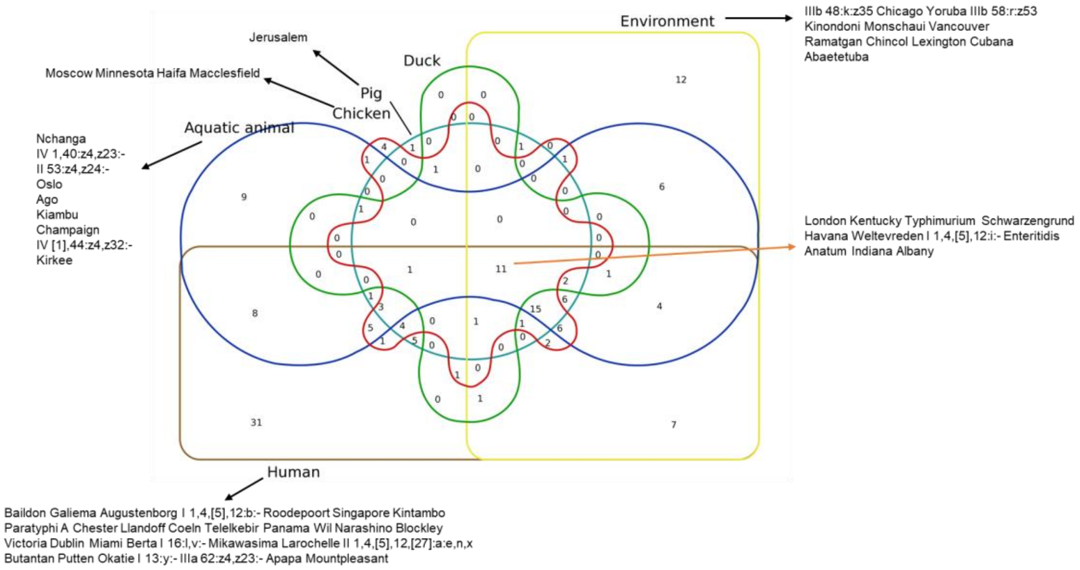

Figure S7. Shared and unique serovars among aquatic animals, chickens, ducks, pigs, humans, and the environment in the CLSGDB v2.

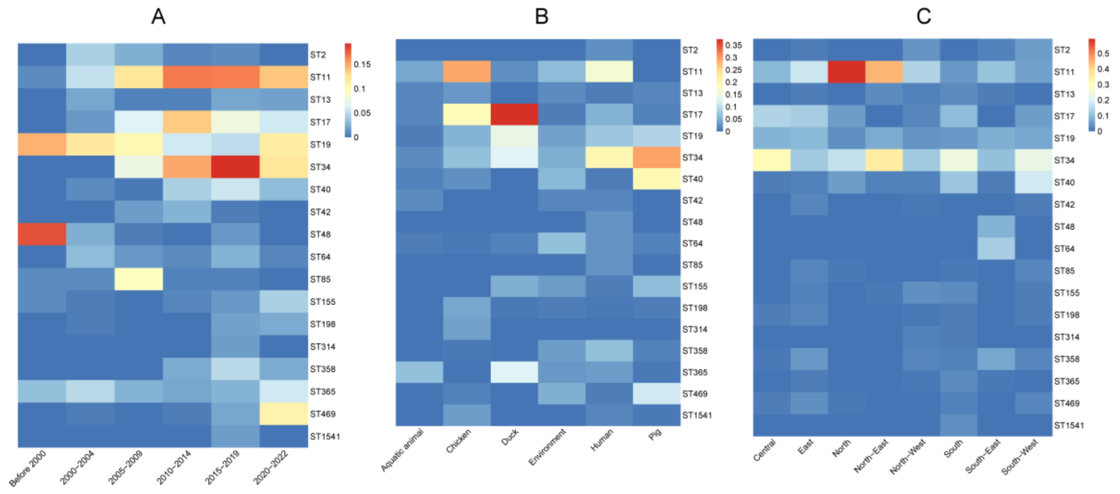

Figure S8. ST prevalence was grouped by sampling periods (A), hosts (B), and regions (C) in the CLSGDB v2.

A total of 295 STs were identified in the CLSGDB v2, and STs that were detected at > 1% prevalence in *Salmonella* genomes in the CLSGDB v2 are shown.

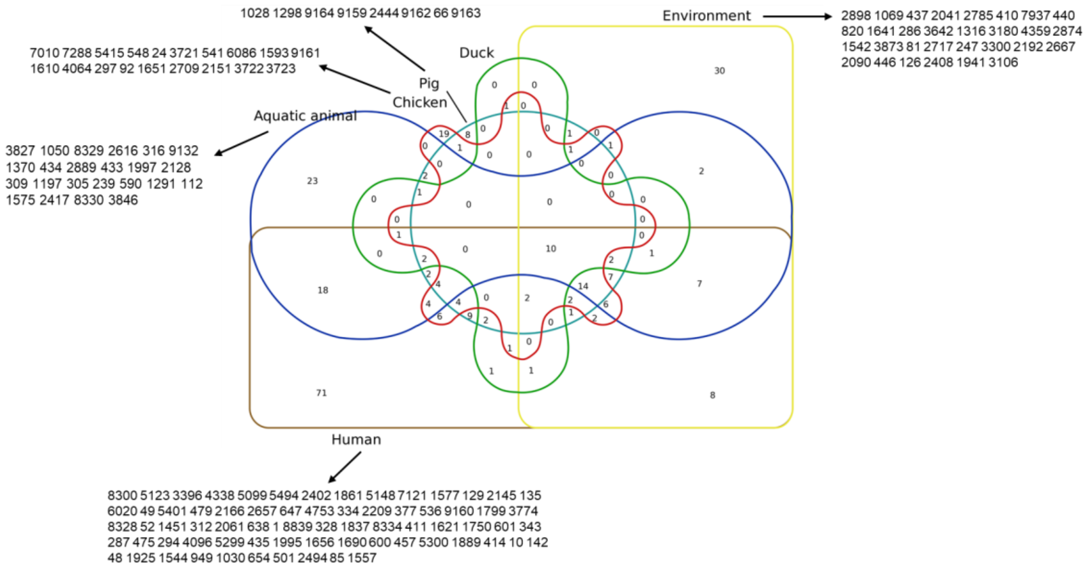

Figure S9. Shared and unique STs among aquatic animals, chickens, ducks, pigs, humans, and the environment in the CLSGDB v2.

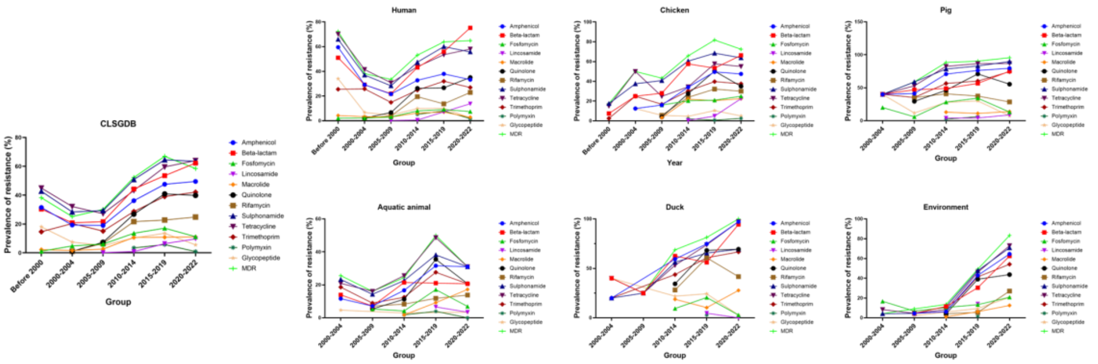

Figure S10. Temporal changes in AMR phenotypes among different isolation sources in the CLSGDB v2.

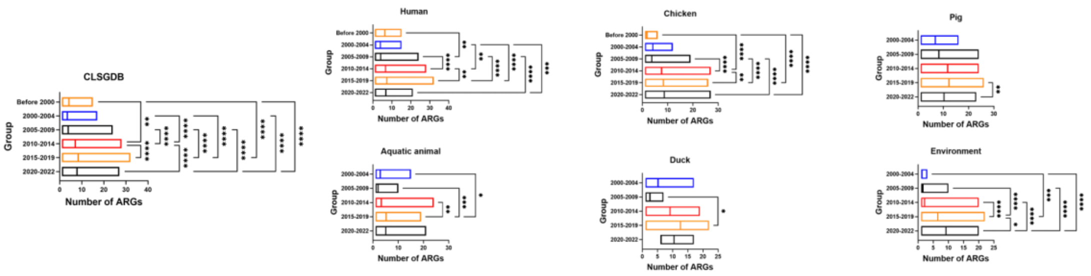

Figure S11. Temporal changes in AMR among different isolation sources in the CLSGDB v2.

Detail of dynamic trends in antimicrobial resistance in the CLSGDB v2.

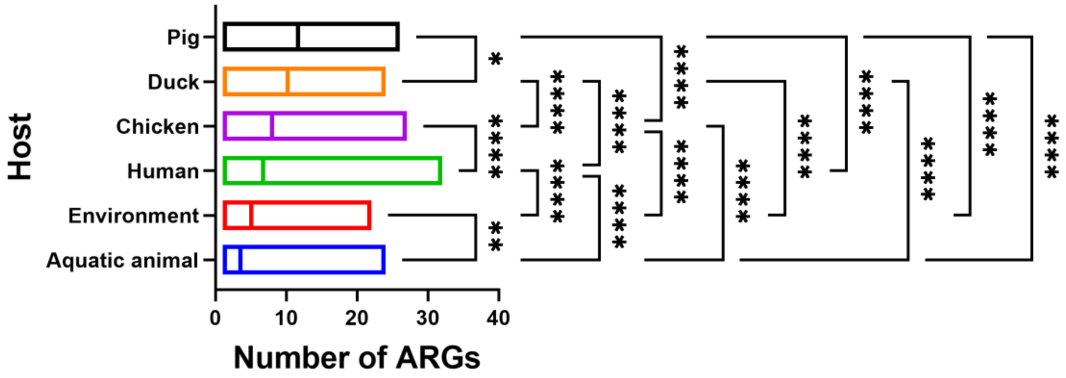

Figure S12. The distribution of the number of ARGs per isolate among humans, the different animal host groups, and the environment in the CLSGDB v2.

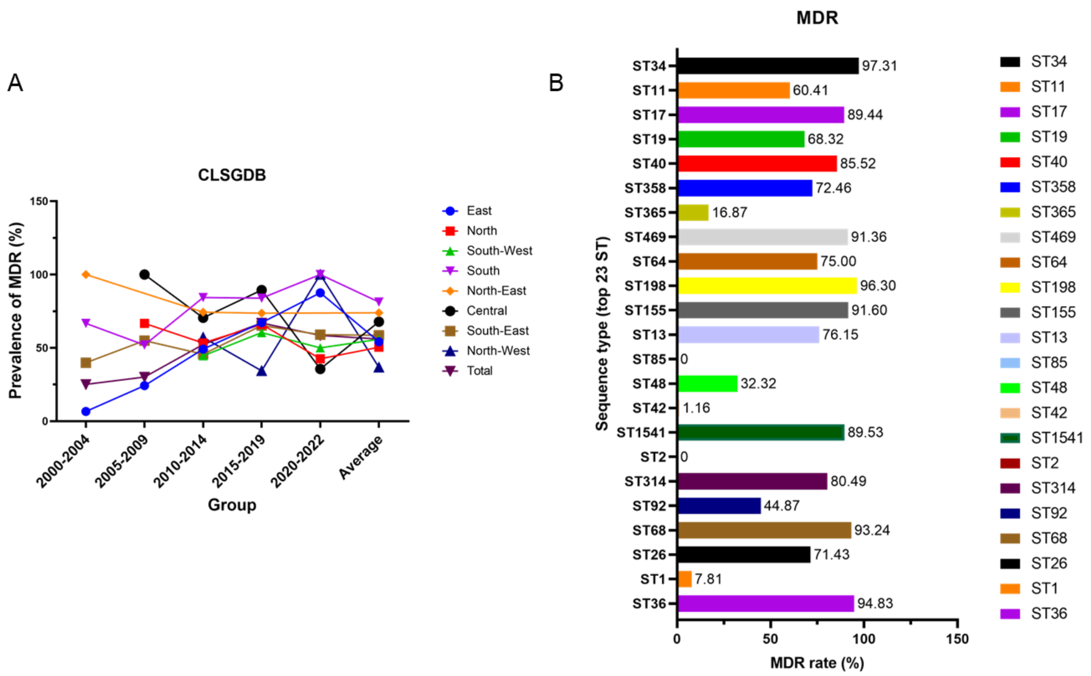

Figure S13. Differences in MDR rates between (A) geographic regions and (B) STs in the CLSGDB v2.

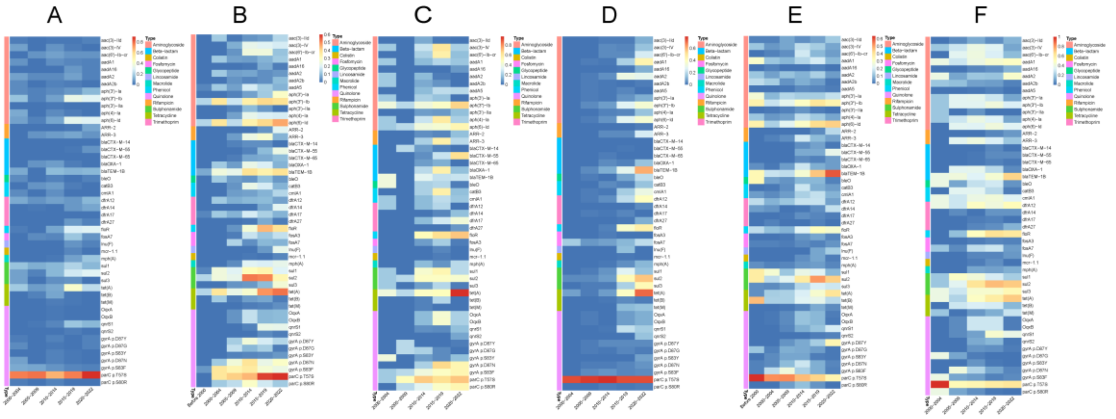

Figure S14. ARG prevalence grouped sampling period among different isolation sources in the CLSGDB v2.

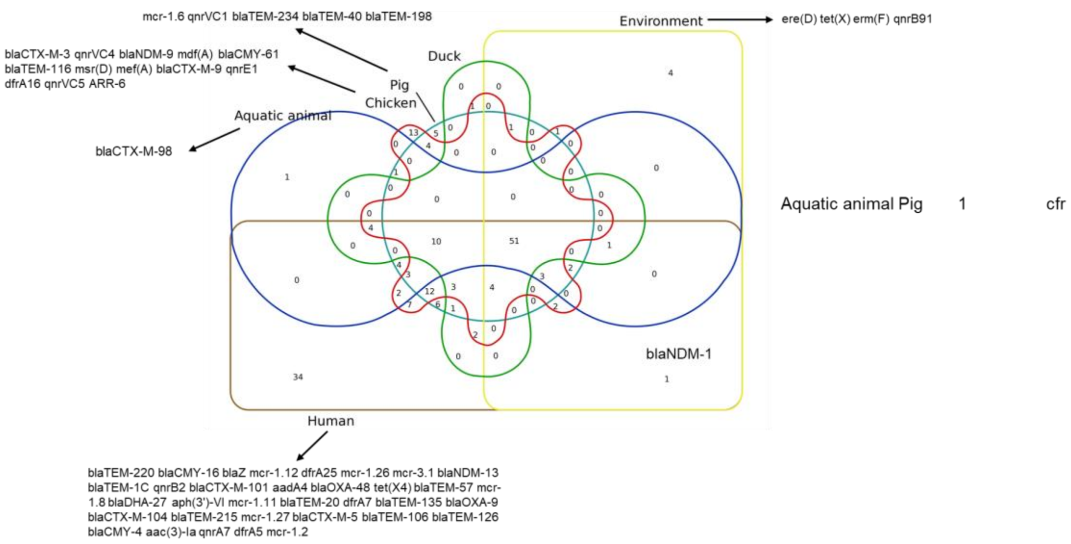

Figure S15. Shared and unique ARGs among aquatic animals, chickens, ducks, pigs, humans, and the environment in the CLSGDB v2.

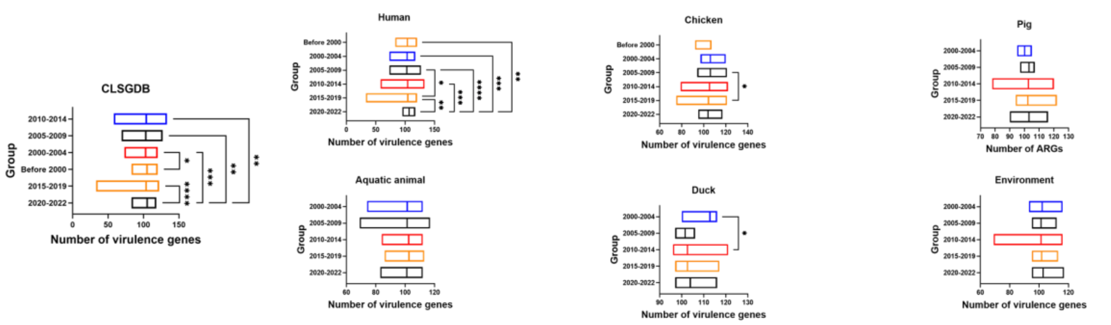

Figure S16. Temporal changes in virulence genes among different isolation sources in the CLSGDB v2.

The mean VGs count for each group is noted above. The ‘*’ on the top represents *P*-values. *: $P < 0.05$; **: $P < 0.01$; ***: $P < 0.001$.

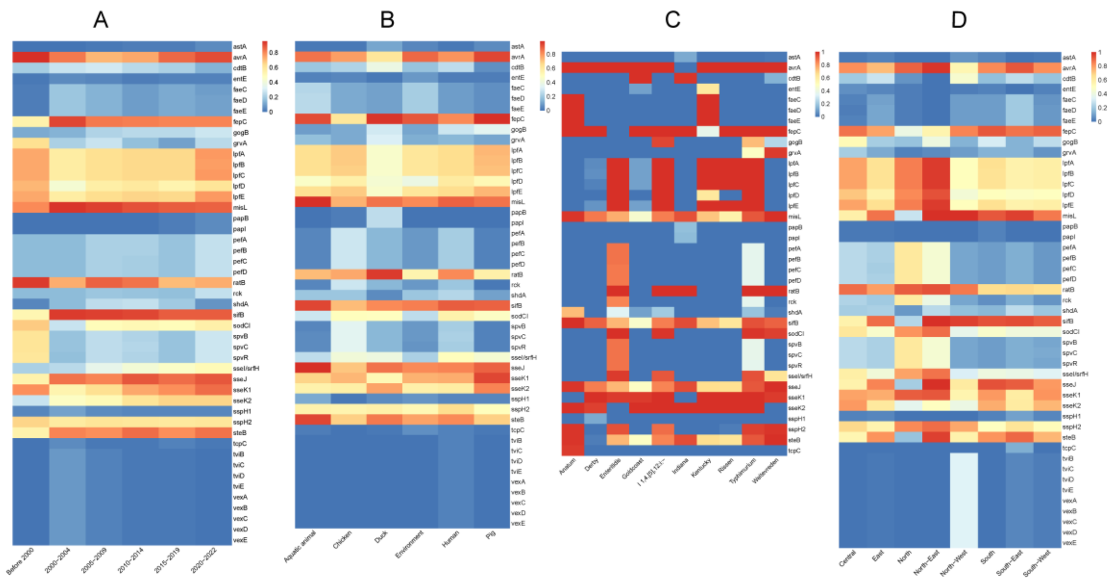

Figure S17. VG prevalence was grouped by sampling periods, hosts, serovars, and regions in the CLSGDB v2.

VGs that were detected from 1% to 86% in *Salmonella* genomes in the CLSGDB v2 are shown.

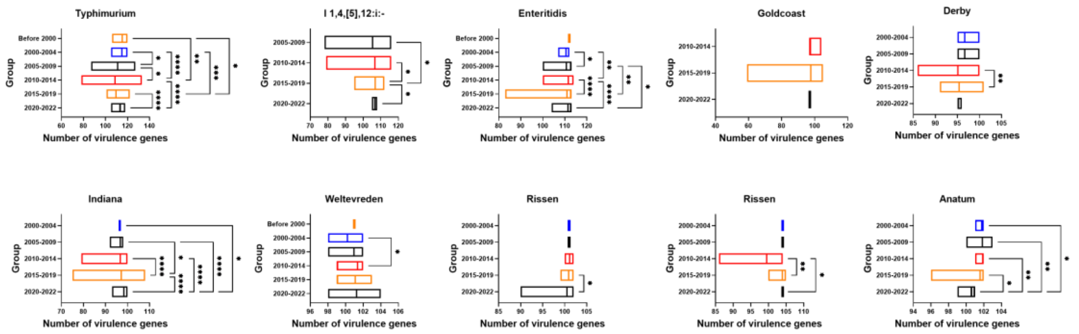

Figure S18. The distribution of the number of virulence genes per isolate among the top 10 serovars in different time groups.

The mean VGs count for each group is noted above. The '*' on the top represents *P*-values. *: $P < 0.05$; **: $P < 0.01$; ***: $P < 0.001$.

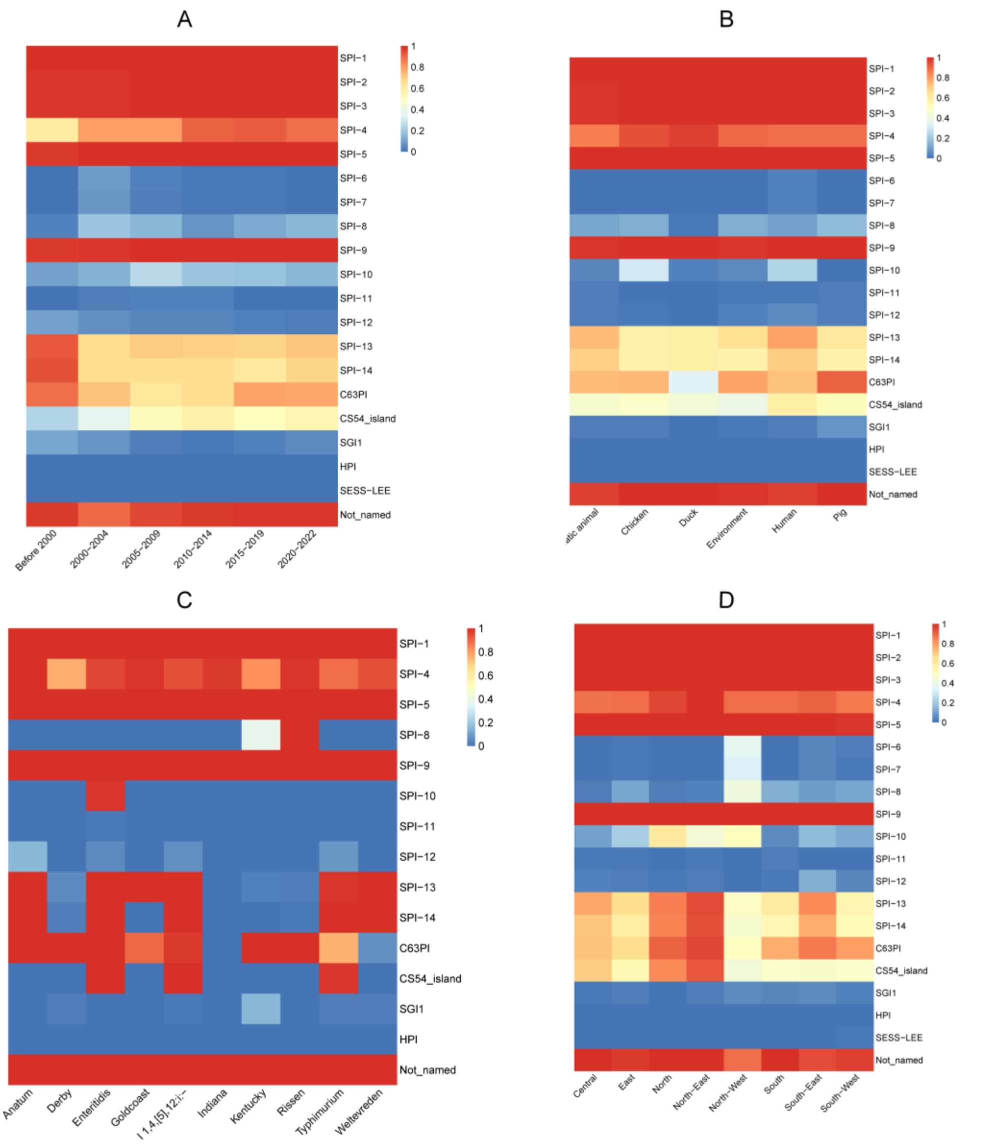

Figure S19. SPI prevalence was grouped by sampling periods, hosts, regions, and serovars in the CLSGDB v2.

SPI2 and SPI3 which were detected 100% in the top 10 serovars of *Salmonella* genomes in the CLSGDB v2 are not shown.

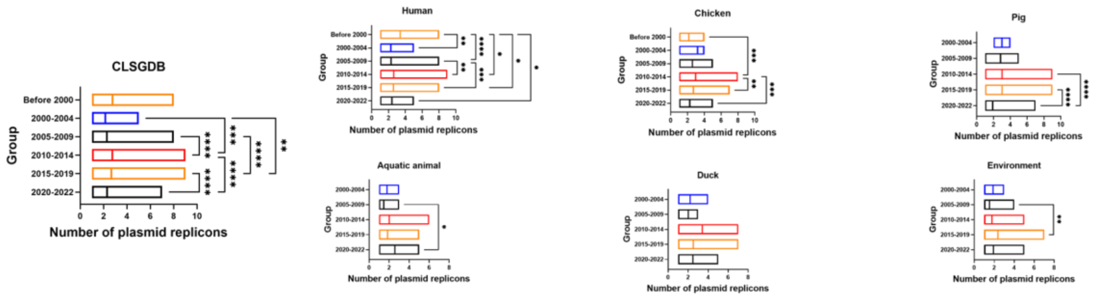

Figure S20. Temporal changes in plasmid replicons among different isolation sources in the CLSGDB v2.

The mean plasmid replicons count for each group is noted above. The ‘*’ on the top represents *P*-values. *: $P < 0.05$; **: $P < 0.01$; ***: $P < 0.001$.

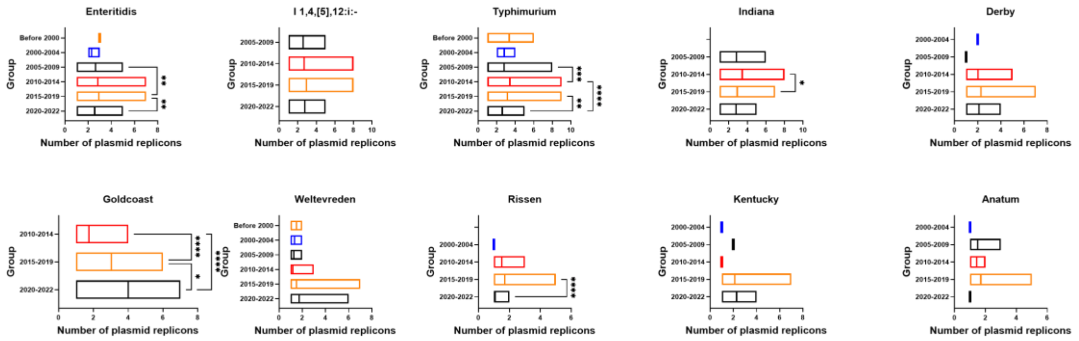

Figure S21. The distribution of the number of plasmid replicon genes per isolate among the top 10 serovars in different time groups.

The mean plasmid replicons count for each group is noted above. The '*' on the top represents *P*-values. *: $P < 0.05$; **: $P < 0.01$; ***: $P < 0.001$.

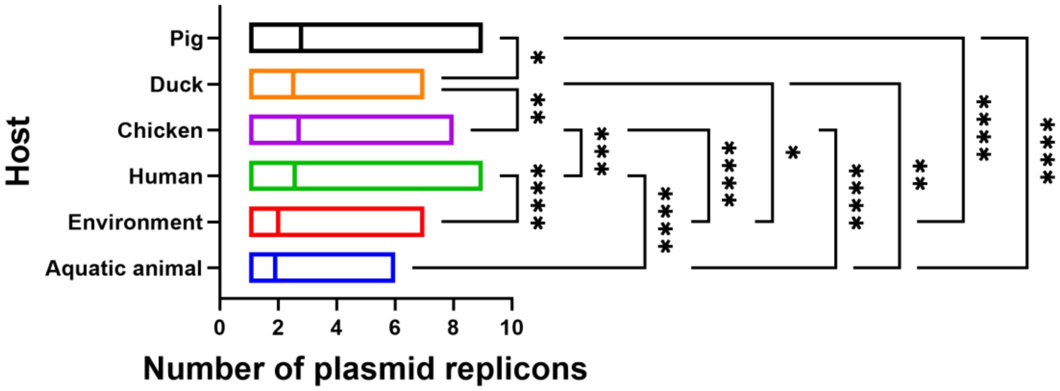

Figure S22. The distribution of the number of plasmid replicons per isolate among different hosts.

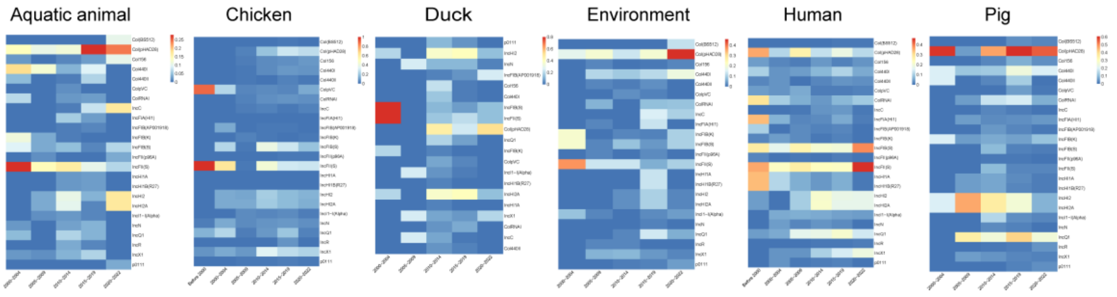

Figure S23. Plasmid prevalence grouped sampling period among different isolation sources in the CLSGDB v2.

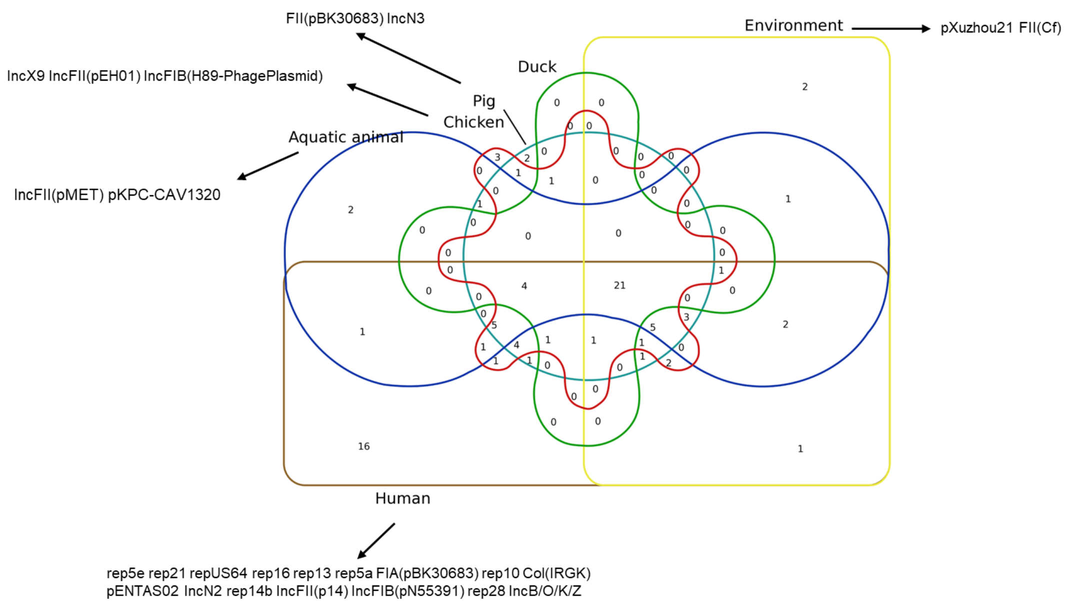

Figure S24. Shared and unique plasmid replicons among aquatic animals, chickens, ducks, pigs, humans, and the environment in the CLSGDB v2.

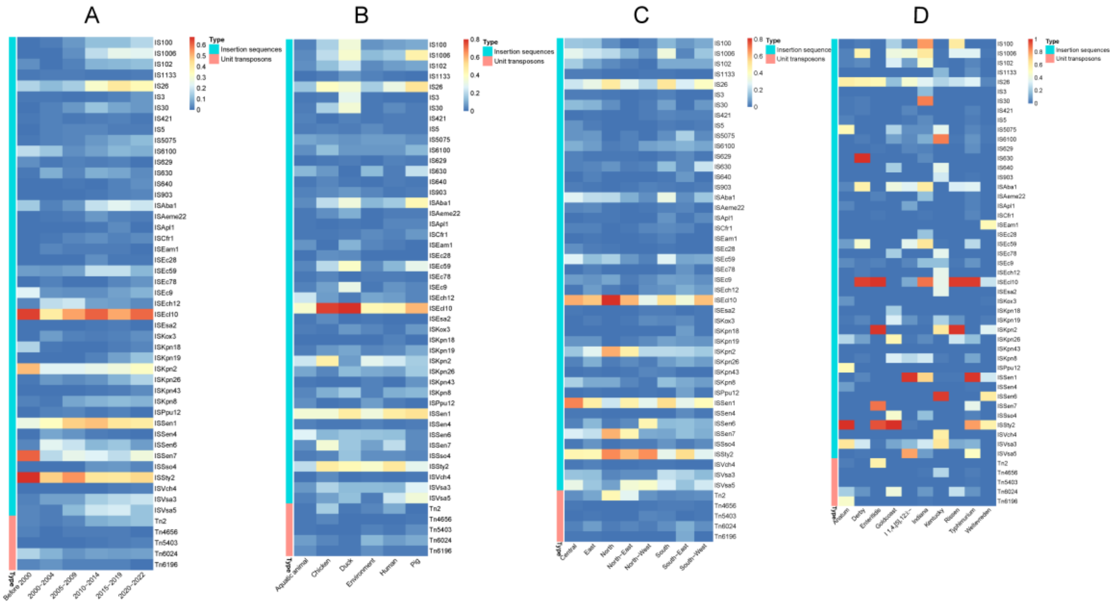

Figure S25. MGE prevalence was grouped by sampling periods (A), hosts (B), serovars (C), and regions (D) in the CLSGDB v2.

A total of 50304 plasmid replicons classified into 1002 types were identified in the CLSGDB v2, replicons that were detected at >1% prevalence (except for MITEEc1 and cn_5129_ISVsa3) in *Salmonella* genomes in the CLSGDB v2 are shown.

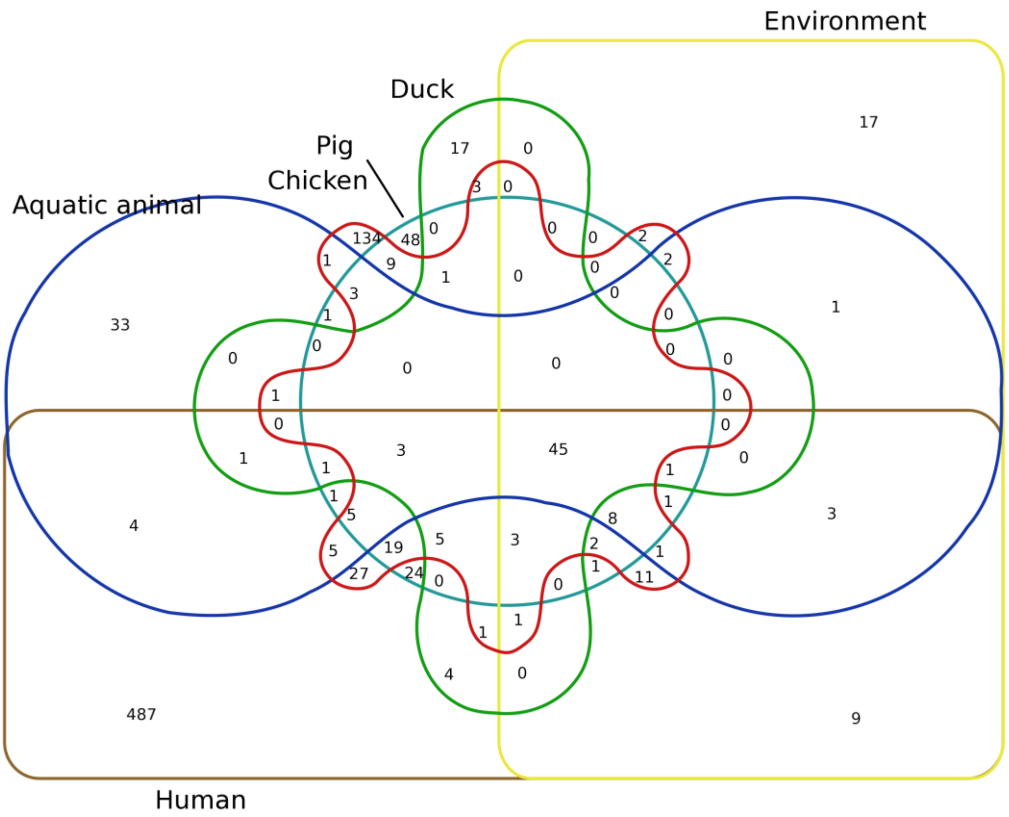

Figure S26. Shared and unique MGEs among aquatic animals, chickens, ducks, pigs, humans, and the environment in the CLSGDB v2.

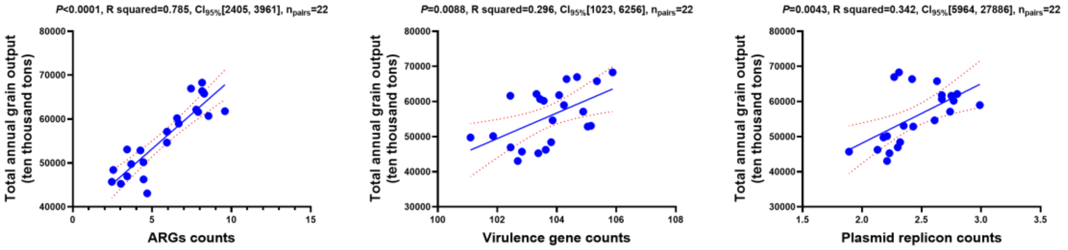

Figure S27. The correlation of annual grain output with ARGs, VGs, and MGEs.

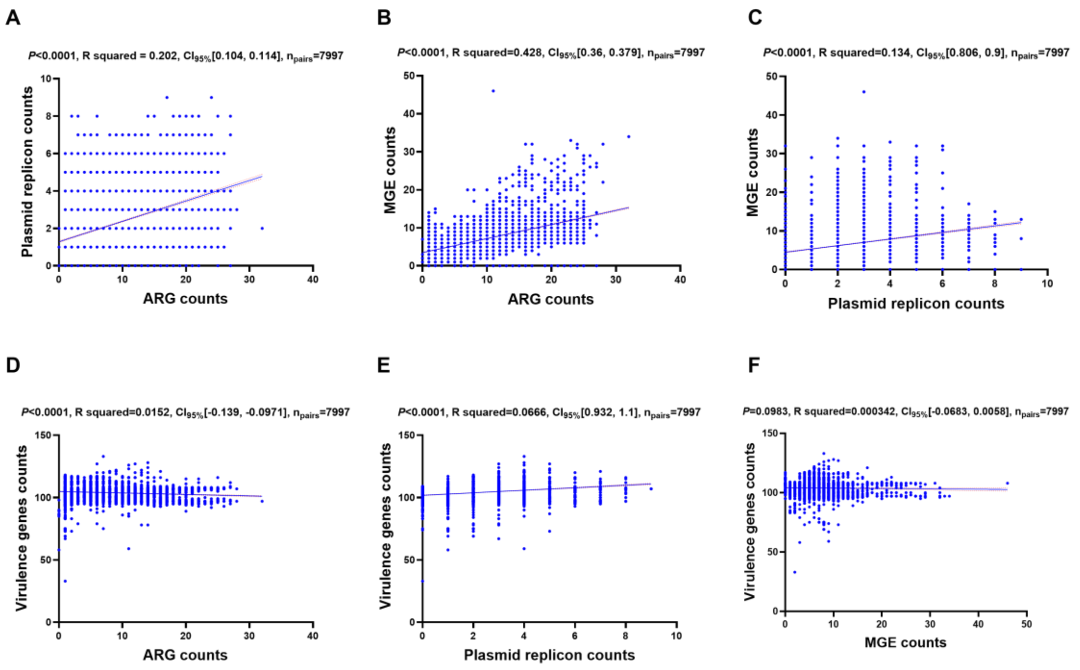

Figure S28. The correlation of mean counts of ARGs, plasmid replicons, MGEs, and VGs detected from genomes in the CLSGDB v2.

A, The correlation between plasmid replicons and ARG counts. B, The correlation between MGEs and ARG counts. C, The correlation between MGEs and plasmid replicons counts. D, The correlation between ARGs and VGs counts. E, The correlation between plasmid replicons and VGs counts. F, The correlation between MGEs and VGs counts.
